# STK33 Phosphorylates Fibrous Sheath Protein AKAP3/4 to Regulate Sperm Flagella Assembly in Spermiogenesis

**DOI:** 10.1016/j.mcpro.2023.100564

**Published:** 2023-05-03

**Authors:** Weiling Yu, Yang Li, Hong Chen, Yiqiang Cui, Chenghao Situ, Liping Yao, Xiangzheng Zhang, Shuai Lu, Li Liu, Laihua Li, Jie Ren, Yueshuai Guo, Zian Huo, Yu Chen, Haojie Li, Tao Jiang, Yayun Gu, Cheng Wang, Tianyu Zhu, Yan Li, Zhibin Hu, Xuejiang Guo

**Affiliations:** 1 State Key Laboratory of Reproductive Medicine and Offspring Health, Nanjing Medical University, Nanjing, China; 2 School of Public Health, Center for Global Health, Nanjing Medical University, Nanjing, Jiangsu, China

**Keywords:** AKAP3, AKAP4, non-obstructive azoospermia, phosphoproteomics, phosphorylation, sperm flagella, STK33

## Abstract

Spermatogenesis defects are important for male infertility; however, the etiology and pathogenesis are still unknown. Herein, we identified two loss-of-function mutations of *STK33* in seven individuals with non-obstructive azoospermia. Further functional studies of these frameshift and nonsense mutations revealed that *Stk33*^*-/KI*^ male mice were sterile, and *Stk33*^*-/KI*^ sperm were abnormal with defects in the mitochondrial sheath, fibrous sheath, outer dense fiber, and axoneme. *Stk33*^*KI/KI*^ male mice were subfertile and had oligoasthenozoospermia. Differential phosphoproteomic analysis and *in vitro* kinase assay identified novel phosphorylation substrates of STK33, fibrous sheath components A-kinase anchoring protein 3 and A-kinase anchoring protein 4, whose expression levels decreased in testis after deletion of *Stk33*. STK33 regulated the phosphorylation of A-kinase anchoring protein 3/4, affected the assembly of fibrous sheath in the sperm, and played an essential role in spermiogenesis and male infertility.

## Highlights

• STK33 has two loss-of-function mutations in the patients of NOA.
• *Stk33*^*-/KI*^ male mice were sterile with defects in sperm formation.
• Fibrous sheath proteins AKAP3/AKAP4 are novel phosphorylation substrates of STK33.
• Sperm from *Stk33*^*−/−*^ male mice have defects in oocyte activation.

## Graphical Abstract

**Figure:**
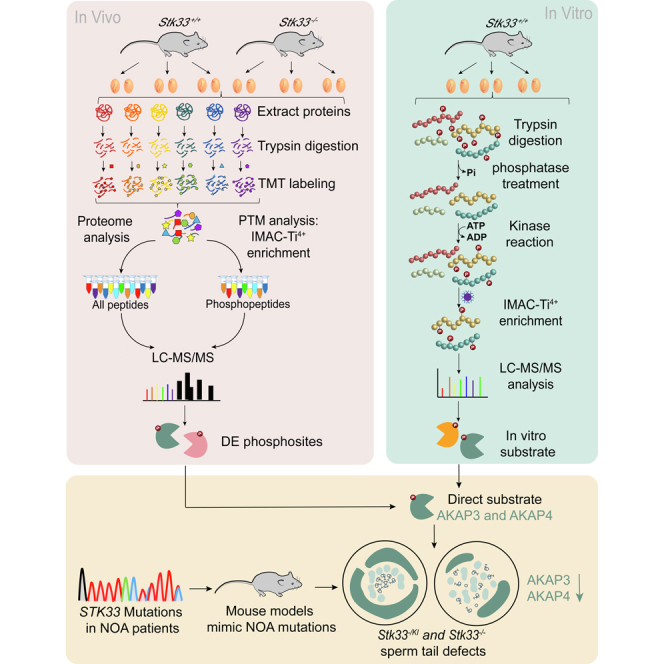

## In Brief

Serine/threonine kinase 33 showed two loss-of-function mutations in the patients of non-obstructive azoospermia, which could cause defects in sperm formation in mouse. Phosphoproteomics analyses revealed that serine/threonine kinase 33 phosphorylates the key components of the fibrous sheath, AKAP3and AKAP4, during mouse sperm flagella assembly. The absence of this kinase affects oocyte fertilization process.

Infertility, defined as the failure to achieve pregnancy after 12 months of regular unprotected sexual intercourse (1), is a growing global issue affecting more than 10% of reproductive-aged couples, half of which could be attributed to male factors (2). Clinically, male infertility usually manifests as oligozoospermia or azoospermia (reduced number of or no sperm), asthenozoospermia (attenuated sperm motility), and teratozoospermia (higher ratio of morphologically abnormal sperm) (3). Azoospermia is the most severe syndrome, which can be divided into obstructive azoospermia and non-obstructive azoospermia (NOA); the latter is characterized as no sperm in the ejaculate due to failure of spermatogenesis (4). Twenty-five percent of patients with NOA can be explained by genetic abnormalities, such as *USP9Y, DMRT1*, and *TEX11*, while the rest are idiopathic (5). Therefore, identifying novel mutations in genes related to spermatogenesis would be beneficial to improve the diagnosis and treatment of NOA patients.

Spermatogenesis, a process by which haploid sperm is generated in the male gonad testis, consists of three major stages: mitosis of spermatogonia, meiosis of spermatocytes, and spermiogenesis of spermatids to form sperm (6). A variety of complex morphological changes occur during spermiogenesis, including acrosome formation, flagella assembly, histone replacement, chromatin condensation, and removal of residual body (7). The flagella formation endows sperm with motility to pass through the female reproductive tract, reaching the ampulla of the uterine tube to encounter and fertilize the ova. The core component of the flagella, axoneme, is an evolutionarily highly conserved “9 + 2” microtubule structure, which is surrounded by outer dense fiber, fibrous sheaths, and mitochondrial sheaths. The fibrous sheath is synthesized from the distal to the proximal end along the axoneme, with the longitudinal column synthesized first and followed by the ribs (8). Correct assembly of all the components is essential for sperm morphology and functions.

Phosphorylation is a well-studied posttranslational modification mediated by various kinases and phosphatases and has been demonstrated to be essential for spermiogenesis (9). For instance, mutations of *AURKC* kinase led to large-headed sperm with multiflagella (10); loss of *Tssk6* affects protamine-histone replacement and male sterility (11). Our systematic analysis of spermatid phosphoproteome undergoing spermiogenesis revealed that serine/threonine kinase 33 (*Stk33*) was an active kinase with enriched substrate phosphorylation sites (12). Deficiency of *Stk33* induced malformed and immotile spermatozoa (13). However, the phosphorylation substrate of STK33 in spermiogenesis is still not known.

Here, we identified two loss-of-function mutations on *STK33* gene in NOA patients, corresponding to the frameshift deletion and truncation of STK33 protein. The study of *Stk33* deletion and knockin mice simulating human mutations showed their critical functions in spermiogenesis. *Stk33*^*KI/KI*^ mice were subfertile, while *Stk33*^*-/KI*^ mice were sterile with spermiogenesis abnormality and a reduced number of sperm, and *Stk33*^*-/KI*^ sperm showed abnormal flagella with defects in axoneme and fibrous sheath. Phosphoproteomic profiling and *in vitro* kinase assay revealed that fibrous sheath proteins, A-kinase anchoring protein 3 (AKAP3) and A-kinase anchoring protein 4 (AKAP4) were phosphorylation substrates of STK33. STK33 is a fibrous sheath regulatory kinase essential for spermiogenesis and male infertility.

## Experimental Procedures

### NOA Patient Population

All methods and experimental protocols on human subjects were approved by research ethics committee of Nanjing Medical University (approval number: [2017]483). Studies in this work abide by the Declaration of Helsinki principles. All patients completed a written informed consent before taking part in this research.

This study included 620 NOA cases recruited from the Nanjing Center of Reproductive Medicine. All infertile male subjects were genetically unrelated Han Chinese men and selected based on andrological examinations, including medical history examination, physical examination, semen analysis, scrotal ultrasound, hormone analysis, karyotyping, and Y chromosome microdeletion screening. Those with a history of cryptorchidism, vascular trauma, orchitis, obstruction of the vas deferens, abnormalities in chromosome number, or microdeletions of the azoospermia factor region on the Y chromosome were excluded from the study. Semen analysis for sperm concentration, motility, and morphology was performed following the World Health Organization (2010) guidelines. Subjects with NOA had no detectable sperm in the ejaculate after evaluating the centrifuged pellet. To differentiate from obstructive azoospermia, only idiopathic azoospermic patients with small and soft testis, normal fructose, and neutral alpha glucosidase in seminal plasma were included in the study. Those with a history of vasectomy were excluded. To ensure the reliability of the diagnosis, each individual was examined twice, and the absence of spermatozoa from both replicate samples was taken to indicate azoospermia. The males in control subjects had fathered one or more healthy children. A 5-ml whole blood sample was obtained from each participant as a source of genomic DNA for further Sanger sequencing analysis.

### Animals

All animals in this study were approved by the Animal Ethical and Welfare Committee of Nanjing Medical University (approval number: IACUC-1707017). All mice were housed in a specific pathogen-free animal facility under standard 12:12 light and dark cycles. All animals in this study were approved by the Institutional Animal Care and Use Committee of Nanjing Medical University.

The KO (*Stk33*^−/−^) mice with frameshift mutation or knockin (*Stk33*^*KI/KI*^) mice were generated using CRISPR/Cas9 genome editing described below. In brief, single-guide RNA (sgRNA) sequences were 5′-ACAAGTGTTT GAGTCGCCTCAGG-3′ and 5′-GTTGATGGTTGACAGTCTTCAGG-3′ for *Stk33*^−/−^ mice and 5′- ATCTGCAAACACAGCAAAGCAGG-3′ for *Stk33*^KI/KI^ mice. sgRNAs were produced and purified using the MEGAshortscript Kit (Ambion, AM1354) and the MEGAclear Kit (Ambion, AM1908), respectively. The fertilized eggs from C57/BL6 mice were subjected to Cas9 mRNA, sgRNA, and/or Donor ssDNA (GATGAGGAGACTGAGCAGAGCGCTGTCTACAGTCCATCTGCAAACACATAAAAGCAGGTAGGAGGGATGGCTGCGACATGCACAGGCTCTGGCACTTTC) injection and transferred into the ampullary-isthmic junction of the oviducts of adult female pseudopregnant recipient mice. DNA from *Stk33*^−/−^ or *Stk33*^KI/KI^ mouse toe was subjected to genotype analysis by PCR using primers (forward: 5′- GCCTCCAAGGAATACCAACTCAAC-3′, reverse: 5′- GCCAAGGACATGAGCA ACTGTG-3′) or (forward: 5′-AGGAGACCAACACAGATGAGGA-3′, reverse:5′-CTCCACTCCAAAGAGCCACTG-3′). The PCR products were subjected to Sanger sequencing.

## Cell Lines

HEK293T cell line was obtained from American Type Culture Collection (ATCC) and has been recently authenticated using short tandem repeat analysis as described in 2012 in ANSI Standard (ASN-0002) by the ATCC standards development organization.

### Histology and Immunofluorescence Analysis

Testes were cut in half and fixed in modified Davidson’s fluid (30% of a 37–40% stock solution of formaldehyde, 15% ethanol, 5% glacial acetic acid, and 50% distilled H_2_O) and embedded in paraffin. Sections were cut at 5-μm thickness.

For H&E staining, sperm from cauda epididymis were fixed in 4% paraformaldehyde for 30 min, washed three times with PBS, then stained with Hematoxylin (Servicebio, G1004) and Eosin (Servicebio, G1002), dehydrated, and mounted. For periodic acid–Schiff (PAS) staining, sections were deparaffinized, rehydrated, stained with PAS’s reagent (Solarbio, G1281), counterstained with hematoxylin (Servicebio, G1004), dehydrated, and mounted. For immunofluorescent staining, sections were subjected to deparaffinization, hydration, and antigen retrieval. Spermatozoa were spread onto microscope slides to air-dry and fixed in 4% paraformaldehyde for 30 min, then washed three times with PBS. The sections of testes or sperm were sequentially blocked with PBS containing 5% bovine serum albumin for 1 h at room temperature, and incubated with primary antibodies overnight at 4 °C, washed in 1 × PBS, incubated with Alexa Fluor 488 (Thermo Fisher Scientific, A21202)– or Alexa Fluor 555 (Thermo Fisher Scientific, A32732)–labeled secondary antibodies for 2 h at room temperature, and counterstained with Hoechst 33342 (Thermo Fisher Scientific, 62249) to label the nuclei. The primary antibodies used here were anti-AKAP3 (Proteintech, 13907-1-AP), anti-AKAP4 (Elabscience, E-AB-65117), and anti-Ac-TUBULIN (Sigma, T6793).

### Electron Microscopy

Ultrastructural examination has been described previously (14). Briefly, 4% (vol/vol) glutaraldehyde-fixed sperm were postfixed with 2% (WT/vol) OsO_4_ and embedded in Araldite. Ultrathin sections (80 nm) were stained with uranyl acetate and lead citrate and analyzed by a transmission electron microscope (JEOL JEM 1010, FEI Tecnai G2, and FEI Tecnai T10).

### Fertility Test and Sperm Analysis

Male mice were housed with WT females of proven fertility for at least 3 months. The litter sizes were recorded continuously. Epididymal sperm were extracted and incubated in human tubal fluid media (Irvine Scientific, 90126) supplemented with 10% fetal bovine serum at 37 °C. Sperm samples were diluted for further examination. The count, motility, and progressive motility of sperm were quantified by computer-assisted semen analysis (CASA) using the IVOS II Sperm Analyzer (Hamilton Thorne). Data were analyzed using an unpaired *t* test with equal SEM (GraphPad Prism 9, https://www.graphpad.com).

### Intracytoplasmic Sperm Injection

The medium used for collecting oocytes from oviducts was Embryomax M2 medium (Sigma, MR-015-D). The subsequent treatments and microinjection were manipulated in Hepes-CZB medium (Nanjing EasyCheck M2750).

To collect oocytes, C57BL6/J female mice, 3- to 4-week-old, were induced to superovulate by intraperitoneal injection of 10 IU pregnant mare serum gonadotropin (PMSG) (Nanjing EasyCheck, M2620) followed by 10 IU human chorionic gonadotropin (hCG) (Nanjing EasyCheck, M2520) 42 to 48 h later. Oocytes were collected from oviducts 12 to 14 h after hCG injection and digested from the cumulus with 0.2% bovine testicular hyaluronidase in M2 medium for 3 min. Oocytes were then transferred into new M2 medium at 37 °C under 5% CO_2_ in air. The semen was squeezed from cauda epididymis and placed in Hepes-CZB medium. A piezo-actuated needle was used to separate the sperm head and tail with the “Clean” mode of PiezoXpert manipulator (Eppendorf) for next micromanipulation.

MII oocytes without or with pre-activation in 10 mM SrCl_2_ in CZB medium for 30 min were exposed to 5 μg/ml cytochalasin B in M2 medium for 5 min. Individual sperm head was injected into a pre-activated oocyte with a piezo-driven pipette. After injection, pre-activated embryos were reactivated by 10 mM SrCl_2_ in CZB medium at 37 °C under 5% CO_2_ for 3 to 5 h, then transferred into a fresh KSOM medium for further culture.

### Protein Sample Preparation, Digestion, and TMT Labeling

Testes from three mice in each WT and *Stk33*^*−/−*^ male groups were subjected to protein extraction, digestion, and tandem mass tag (TMT) labeling. In short, proteins in testicular tissues were extracted by protein extraction buffer (8 M urea, 75 mM NaCl, 50 mM Tris, pH 8.2, 1% (vol/vol) EDTA-free protease inhibitor, 1 mM NaF, 1 mM β-glycerophosphate, 1 mM sodium orthovanadate, 10 mM sodium pyrophosphate, 1 × cocktail), reduced, trypsin digested, and desalted by Sep-Pak column from Waters Co (Milford) as described previously (14, 15). The reagents were purchased from Sigma-Aldrich.

For TMT labeling, purified peptides were reconstituted in 200 mM triethylammonium bicarbonate and labeled using TMT 6-plex according to the manufacturer’s instructions. All labeled peptide samples were combined, purified using an OASIS HLB Vac cartridge (Waters), and lyophilized.

### High-pH Reverse Phase Fractionation

The TMT-labeled peptides were fractionated by a high-pH reverse phase column as previously described (16). Briefly, for protein quantification, 30 μg TMT-labeled peptide mixture was fractionated using ACQUITY UPLC M-Class with XBridge BEH C18 column (300 μm × 150 mm, 1.7 μm; 130 Å, Waters). Buffer A (10 mM ammonium formate, pH 10) and buffer B (100% acetonitrile (ACN)) were employed under a 128 min gradient (3% buffer B for 14 min, 3%–8% B for 1 min, 8%–29% B for 71 min, 29%–41% B for 12 min, 41%–100% B for 1 min, 100% B for 8 min, and 100%-3% B for 1 min followed by 20 min at 3% B). Thirty fractions were collected using nonadjacent pooling scheme and then dried with a SpeedVac concentrator.

For phosphoproteomic quantification, 3 mg TMT-labeled peptide mixture was fractionated using Agilent 1260 system with XBridge BEH300 C18 column (10 × 250 mm, 5 μm; Waters). Ten fractions were collected by using a nonadjacent pooling scheme within a 22 min gradient of 0%–12% buffer B (5 mM ammonium formate/90% ACN, pH 10 for 1.7 min, 12%–32% B for 10.6 min, 32%–37% B for 0.7 min, 37%–48% B for 2.6 min, and 48%–70% B for 2.4 min, followed by 4 min at 70% B). The 10 fractions were then dried by vacuum concentration for further phosphopeptide enrichment.

### *In vitro* Kinase Assay

*In vitro* kinase assay was performed as described previously (17), with minor modifications. Proteins from testicular tissues were extracted in protein extraction buffer as above. Proteins were digested by trypsin, and 200 μg peptides were resuspended in 200 μl of phosphatase incubation buffer (5 mM Tris pH 8.2, 10 mM NaCl, 1 mM MgCl2, and 0.1 mM DTT) and treated by 50 units of alkaline phosphatase (Sigma, P0114) at 37 °C for 3 h, followed by phosphatase deactivation by heating at 75 °C for 5 min. After desalination and lyophilization, the dephosphorylated peptides were resolubilized by 200 μl kinase reaction buffer (10 mM MgCl2, 1 mM DTT, 1 mM ^18^O-ATP in 50 mM Tris–HCl, pH 7.5) containing 0.5 μg purified STK33 kinase or STK33 kinase-dead at 30 °C for 1 h with shaking. Reactions were quenched by adding 0.4% TFA to pH <3, and the samples were desalted with a 10 mg OASIS HLB Vac cartridge (Waters) and dried for further phosphopeptide enrichment and mass spectrometry analysis.

### Phosphopeptide Enrichment

TMT-labeled phosphopeptides and phosphopeptides from *in vitro* kinase assay were enriched through Ti-immobilized metal affinity chromatography (IMAC, JKchemical) (12, 15, 18). In brief, peptides were dissolved in loading buffer (80% ACN, 6% TFA), incubated with IMAC beads for 30 min at room temperature, and washed with wash buffer I (50% ACN, 200 mM NaCl, 6% TFA) and II (30% ACN, 0.1% TFA) for 30 min, respectively. Then, the beads were eluted by elution buffer (10% NH_4_OH) for 15 min. The eluates of phosphopeptides were dried and desalted by C18 StageTips.

### Mass Spectrometric Data Acquisition

For LC-MS/MS analyses, peptides or phosphopeptides were resuspended in 0.1% formic acid (FA) and analyzed using an LTQ Orbitrap Fusion Lumos mass spectrometer (Thermo Finnigan) coupled to the Easy-nLC 1200. The trap column (75 μm × 2 cm, Acclaim PepMap100 C18 column, 3 μm, 100 Å; DIONEX) effluent was transferred to a reverse-phase microcapillary column (75 μm × 25 cm, Acclaim PepMap RSLC C18 column, 2 μm, 100 Å; DIONEX). A 95-min linear gradient (3% to 5% buffer B for 5 s, 5% to 15% buffer B for 40 min, 15% to 28% buffer B for 34.8 min, 28% to 38% buffer B for 12 min, 30% buffer to 100% buffer B for 5 s, and to 100% buffer B for 8 min) was applied using the following buffer: 0.1% FA (buffer A) and 80% ACN, 0.1% FA (buffer B). The Orbitrap Fusion Lumos mass spectrometer was operated in the data-dependent mode. A full survey scan was obtained for the *m/z* range of 350 to 1500. For both protein expression and phosphorylation quantification, the quadrupole isolation window was set to 1Th, and the resolution of high-energy collision-induced dissociation tandem mass spectrometry (HCD MS/MS) was 15,000.

### Bioinformatics Analysis

TMT-based mass spectrometry raw files were searched against the mouse protein sequences from the Universal Protein Resource (UniProt, 2018.07.18; 61,655 entries) database by MaxQuant software (version 1.6.2.10, https://www.maxquant.org), and for *in vitro* kinase assay, MaxQuant software (V1.6.5.0) was used to search the Universal Protein Resource (UniProt, 2020.01.15; 63,628 entries). MaxQuant uses individual mass tolerances for each peptide, whereas the mass tolerances for initial maximum precursor were set to 20 ppm in the first search and 4.5 ppm in the main search, and the fragment mass tolerance was set to 20 ppm. Carbamidomethyl (C) on cysteine, TMT reagent adducts on lysine, and peptide amino termini were fixed modifications for TMT data. Variable modifications included oxidation (M) and acetylation (protein N-term). A dynamic modification of +79.996 Da on serine, threonine, and tyrosine residues in TMT data was recognized as a phosphoryl group. A dynamic modification of +85.996 Da on serine, threonine, and tyrosine residues in *in vitro* kinase assay data was recognized as a heavy phosphoryl group. False discovery rate (FDR) cut-offs were set to 0.01 for proteins, peptides, and phosphorylation sites.

Phosphorylation sites were filtered with localization probability >0.75, and only type I phosphorylation sites (singly phosphorylated peptides) were subjected to downstream analysis. The proteins may be regulated at both protein expression and phosphorylation levels after *Stk33* deletion, and the decreased expression of a protein can result in decreased absolute phosphorylation level of the protein, even without kinase regulation. To better reflect the phosphorylation regulation by kinase activity in *Stk33*^*−/−*^ testes, the level of each phosphorylation site was normalized against the abundances of the corresponding proteins for the TMT-based phosphoproteomic data. Statistical significance was determined using the unpaired two-tailed Student’s *t* test, and *p* <0.05 was considered as significant.

To obtain an overview of the function of proteins identified by our proteomic analysis, we applied Gene Ontology (GO) enrichment analysis using “clusterProfiler” package in *R*. The network of biological process was constructed by Cytoscape (Version 3.9.1, https://cytoscape.org). *p* values for GO categories were adjusted considering FDR using Benjamini–Hochberg method. And FDR <0.05 was considered significant. MoMo (Version 5.4.1, https://meme-suite.org/meme/tools/momo) was used in motif analysis, and the top2 were selected as the analysis objects.

### Experimental Design and Statistical Rationale

For differential phosphoproteomic analysis, testes from three mice in each WT and *Stk33*^*−/−*^ male group were subjected to protein extraction, digestion, and TMT 6-plex labeling. The TMT-labeled peptides were fractionated by a high-pH reverse phase. For phosphorylation quantification, a total of ten fractions were collected using Agilent 1260 system and subjected to Ti-IMAC enrichment for phosphopeptides. For protein quantification, a total of 30 fractions were collected using ACQUITY UPLC M-Class system. All quantification results are presented as the mean ± SEM values. The statistical significance of the differences was determined using unpaired two-tailed Student’s *t* test or one-way ANOVA. Post hoc test was performed by Turkey’s multiple comparisons test. Each experiment was performed at least three times, and *p* <0.05 was considered significant.

### Plasmids Construction and Prokaryotic Expression

Full-length complementary DNA (cDNA) carrying *Stk33*, *Akap3*, or *Akap4* was ligated to the pcDNA3.1(−) vector by homologous recombination. The mutation sites were designed on the primers to amplify the mutant form of *STK33*, and the hemagglutinin (HA) and FLAG tags were designed on the primers to obtain the tagged plasmids. The *Stk33* gene in 6∗His-STK33 was chemically synthesized by GenScript company and inserted into the pet28a (+) vector. The primers used to amplify each gene are listed in supplemental Table S5.

The BL21 competent *Escherichia coli* was transfected with 6∗His-STK33 or 6∗His-STK33 KD (K140M) plasmids to produce large amounts of proteins, which were then purified by nickel-nitrilotriacetic acid agarose (Ni-NTA agarose, Qiagen 30210), which is an affinity chromatography matrix for purifying recombinant proteins carrying a His tag, under native conditions. The protein yield was determined by NanoDrop spectrophotometry and Coomassie Brilliant Blue staining.

### Co-immunoprecipitation

HEK293T cells were transfected with pcDNA3.1 eukaryotic expression plasmids using Xfect transfection reagent (Vazyme, T101). Two days after transfection, cells were lysed with Pierce immunoprecipitation Lysis Buffer (Thermo Fisher Scientific, 87787) supplemented with 1% (vol/vol) protease inhibitor mixture (Bimake, B14001) for 40 min at 4 °C and then clarified by centrifugation at 12,000*g* for 20 min. The extracted proteins were incubated with primary antibodies overnight at 4 °C. Next, 50 μl of protein A/G magnetic beads (Thermo Fisher Scientific, 88802 and 88837) or G1 Affinity Resin (GenScript, L00432) was added to each incubation sample for 2 h at room temperature or overnight at 4 °C. Then, the beads were washed three times with 1 × PBST (0.5% TritonX-100), after which co-immunoprecipitated proteins were eluted by standard 5 × SDS sample buffer and heated for 10 min at 95 °C. The co-immunoprecipitated proteins were subjected to Western blot analysis.

### Western Blotting

Proteins of cultured cells and testis tissues were extracted using a universal protein extraction lysis buffer (Beyotime, P0013C) containing a protease inhibitor cocktail (Sigma, P8340). The denatured proteins were separated on 10% or 12.5% SDS-polyacrylamide gels (Epizyme, PG112 and PG113) and transferred to a polyvinylidene difluoride membrane (Bio-Rad, 1620177) for the immunoblot analysis. The primary antibodies used in Western blotting were anti-STK33 (Proteintech, 12857-1-AP), anti-GFP (Abways, AB0005), anti-β-TUBULIN (Abclonal, AC-021), anti-HA (Sigma, H6908), anti-FLAG (MBL, PM020), anti-AKAP3 (Proteintech, 13907-1-AP), anti-AKAP4 (Elabscience, E-AB-65117), anti-GAPDH (Abways, AB0036), anti-His (Abways, AB0002), and anti-Phosph-(Ser/Thr) Phe (Abcam, ab17464).

## Results

### Identification of *STK33* Gene Mutations in NOA Patients

**Fig. 1:**
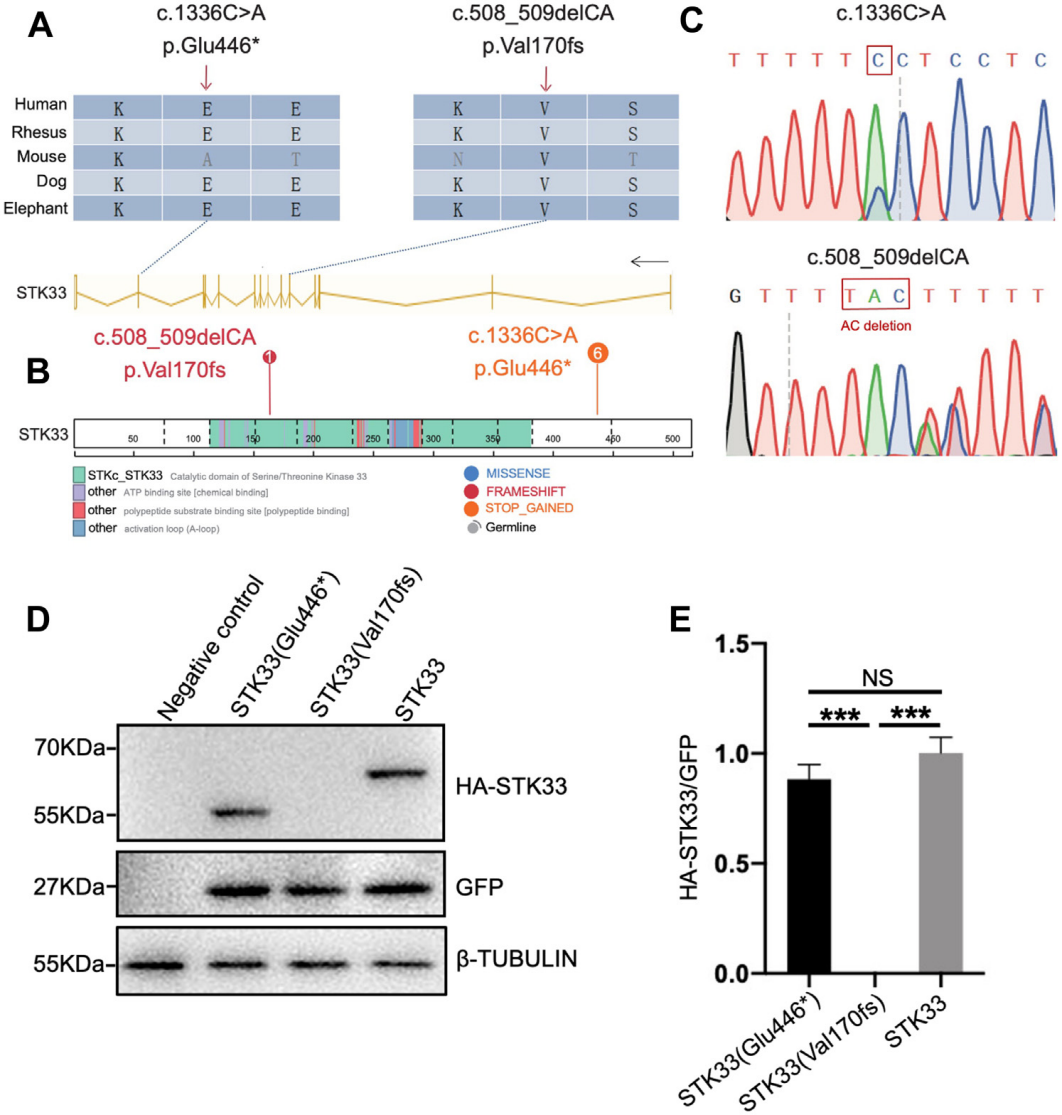
**Identification of loss-of-function mutations in *STK33* from patients with NOA.***A*, sequence similarity of *STK33* mutant sites in various organisms. *B*, schematic representation of the STK33 protein with known protein domains is indicated. The *orange* mutations represent stop-gained mutations and *red* ones represent frameshift mutations. C, sequencing graphs of *STK33* mutations identified from NOA patients. *D* and *E*, Western blots (*D*) and statistics analysis (*E*) of the overexpression of *STK33* mutant proteins in 293T cells. (N = 3). All data are presented as mean ± SEM and analyzed by one-way ANOVA. NS, not significant; ∗∗∗*p* < 0.001. NOA, non-obstructive azoospermia; Stk33, serine/threonine kinase 33.

*STK33* (NM_030906) is localized on chromosome 11 and contains 14 exons encoding a predicted 514-aa protein (Q9BYT3). To evaluate the association of the *STK33* functional variations with human male infertility, we sequenced the 16 exons and the intron boundaries of *STK33* gene in a cohort of 620 patients with NOA. All patients underwent semen analyses at least on three different occasions, and those with a history of orchitis, obstruction of vas deferens or endocrine disorders were excluded. We identified two different pathogenic variants in *STK33* in seven unrelated individuals from the cohort. The nonsense mutation (c.1336C>A, p.Glu446∗) was identified in six subjects and affected a conserved residue in exon 13 of the *STK33* gene. The other mutation was small frameshift indels (c.508_509delCA, p.Val170fs) located in exon 6 (Fig. 1, *A*–*C* and Table 1). In addition, both variants were found in the gnomAD database with very low prevalence in the general population (MAF = 7.25e-5 and 0, respectively, Table1). All these mutations are predicted to produce no protein or truncated nonfunctional proteins. Thus, to evaluate the effect of these mutations on STK33 protein expression, we introduced the mutation sites into the human *HA-STK33-IRES-GFP* construct and overexpressed them in HEK293T cells. It turned out that the protein with c.1336C>A was expressed in a truncated form with an equivalent expression level compared with WT full-length STK33, while c.508_509delCA protein could not be detected (Fig. 1, *D* and *E*). In summary, the two NOA-related mutations (c.1336C > A and c.508_509delCA) could lead to a truncated form or deletion of STK33 protein, respectively, indicating loss-of-function of STK33 is associated with male infertility.

**Table 1:** Loss-of-function mutations in the STK33 gene

Location	Mutation type	Protein alteration	CADD^a^	Affect Allele	Allele frequency in populations				
					Cases	gnomAD All	gnomAD East	1KG All	1KG East
Chr11:8435050 C>A	Nonsense	p.Glu446∗	40	Het	0.0048	3.23E-05	0.000617	0	0
Chr11:8483400 TAC>T	Frameshift	p.Val170fs	NA	Het	0.0008	0	0	0	0

Abbreviation: CADD, Combined Annotation Dependent Depletion; Het, heterozygous; NA, not available.

N_Case_=620.

a The function of the mutations are predicted by Combined Annotation Dependent Depletion tool.

### *Stk33*^*KI/KI*^ Mice Were Subfertile With Decreased Count and Motility of Sperm

**Fig. 2:**
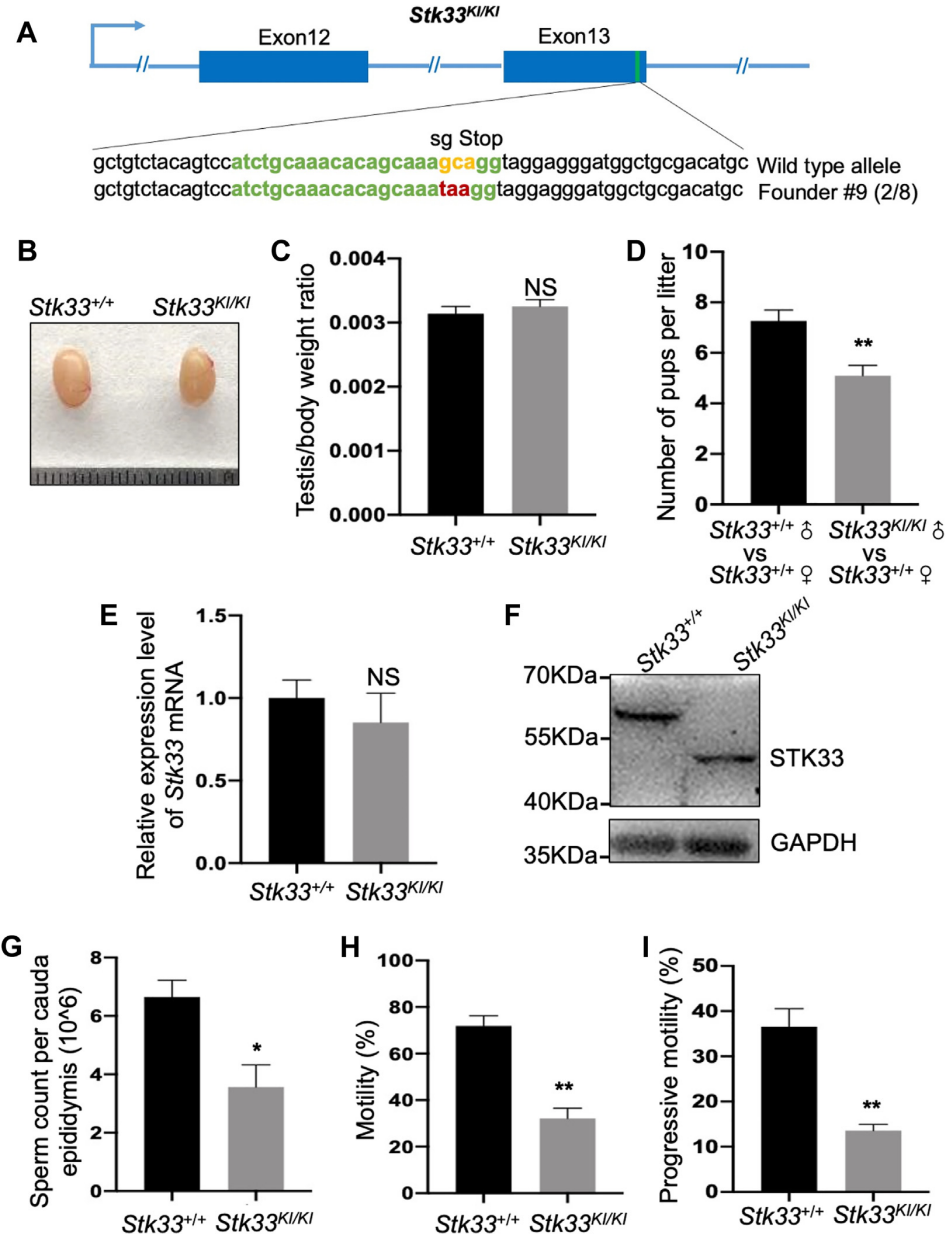
***Stk33***^***KI/KI***^**male mice were subfertile and the count and motility of sperm reduced.***A*, schematic diagram of CRISPR/Cas9 targeting strategy. The sgRNAs were designed within exon 13 of *Stk33*, and stop codon was detected in *Stk33*^*KI/KI*^ mice by Sanger sequencing. *B*, photo of WT and *Stk33*^*KI/KI*^ testes. *C*, average testis weight/body weight in WT and *Stk33*^*KI/KI*^ mice. (N = 3). *D*, average number of pups per litter from WT and *Stk33*^*KI/KI*^ male mice. (N = 6). *E*, *Stk33* mRNA expression levels in WT and *Stk33*^*KI/KI*^ testes. (N = 3). *F*, Western blot assay indicated that truncated STK33 protein was expressed in *Stk33*^*KI/KI*^ mice. *G*, sperm count in single cauda epididymis from WT and *Stk33*^*KI/KI*^ mice. (N = 3). *H* and *I*, average percentages of motile spermatozoa (*H*) and progressively motile spermatozoa (*I*) from WT and *Stk33*^*KI/KI*^ mice. (N = 3). All data are presented as mean ± SEM and analyzed by unpaired 2-tailed Student’s *t* test. NS, not significant; ∗*p* < 0.05, ∗∗*p* < 0.01. Stk33, serine/threonine kinase 33.

In order to further explore the function of STK33 (Glu446∗) in spermatogenesis, a mouse model *Stk33*^*KI/KI*^ that mimics the human nonsense mutation (Glu446∗) was constructed (referred to as *Stk33*^*KI/KI*^ mice) (Fig. 2*A*). Phenotypic analysis indicated that *Stk33*^*KI/KI*^ mice had the natural testicular appearance and normal testis/body weight ratio, with no decrease of mRNA and protein expression levels of *Stk33* (Fig. 2, *B*, *C*, *E* and *F*). Interestingly, *Stk33*^*KI/KI*^ mice displayed a subfertile phenotype with a decreased litter size (Fig. 2*D*). CASA assay showed that the count, motility, and progressive motility of *Stk33*^*KI/KI*^ sperm significantly decreased compared to WT controls (Fig. 2, *G*–*I* and supplemental Movie S1). We also performed a morphology analysis of *Stk33*^*KI/KI*^ sperm from the caudal epididymis. The results showed that *Stk33*^*KI/KI*^ sperm exhibited a similar ratio of abnormal sperm to the control sperm (supplemental Fig. S1, *A* and *B*). In addition, *Stk33*^*KI/KI*^ testis underwent normal spermatogenesis with normal seminiferous stages as shown by PAS staining of testicular sections (supplemental Fig. S1*C*). As the *Stk33*^*KI/KI*^ sperm had decreased motility and progressive motility, we further performed an electron microscopic analysis of *Stk33*^*KI/KI*^ sperm. We found that *Stk33*^*KI/KI*^ sperm showed normal mitochondrial sheath, “9 + 2” microtubule, and fibrous sheath similar to that of the control sperm (supplemental Fig. S1*D*). Thus, *Stk33*^*KI/KI*^ mice displayed male subfertility with reduced sperm count and motility.

### *Stk33*^*-/KI*^ Mice Were Sterile With Decreased Number and Structural Abnormalities of Sperm

As *STK33* also showed frameshift mutation in NOA patients, we used CRISPR-Cas9 technology to generate *Stk33* frameshift mutation in *Stk33*^*−/−*^ mice (supplemental Fig. S2*A*). We found that STK33 protein was not detected in the *Stk33*^*−/−*^ testis (supplemental Fig. S2*B*), indicating null mutation and knockout of *Stk33*, and the male *Stk33*^*−/−*^ mice were infertile (supplemental Fig. S2*C*). The number of spermatozoa was significantly reduced in *Stk33*^*−/−*^ mice compared to that of the WT control mice (supplemental Fig. S2*D*). Morphological analysis of *Stk33*^*−/−*^ sperm revealed abnormalities in 72.0% of heads and 100% of tails, including irregularly shaped sperm heads, short, stumped, coiled, and absent flagella, significantly higher than the control sperm (supplemental Fig. S2, *E* and *F*). To characterize the exact stages of abnormalities during spermiogenesis, we performed PAS staining of the testis. The results showed that the *Stk33*^*−/−*^-elongated spermatids exhibited deformities in the head from IX-X stages, and *Stk33*^*−/−*^ spermatids only showed scattered tail structures at IV-VIII stages, significantly fewer than that of the control spermatids neatly arranged on the lumen surface (supplemental Fig. S2*G*). We also analyzed the ultrastructure of *Stk33*^*−/−*^ sperm from cauda epididymis using a transmission electron microscope. The *Stk33*^*−/−*^ sperm displayed the consistent aberrant nuclei and disorganized flagella including misaligned mitochondria, disorganized outer dense fiber and axoneme, and fragmented fibrous sheath structures comprising longitudinal and lateral columns in the principal piece (supplemental Fig. S3*A*). These data demonstrated that *Stk33* was indispensable for spermiogenesis and male fertility, consistent with previous reports (13).

**Fig. 3:**
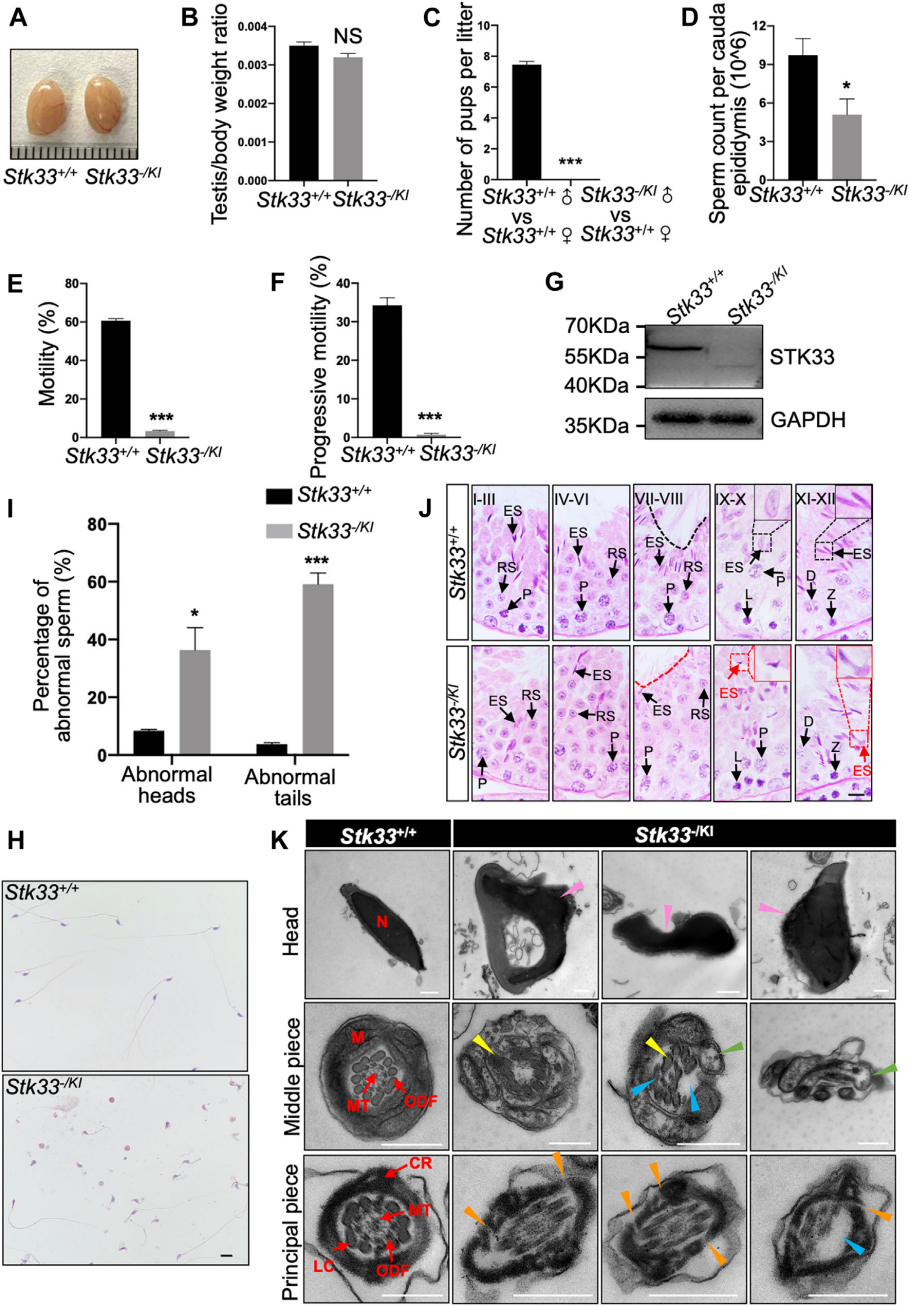
***Stk33***^***-/KI***^**male mice were sterile and had a reduced number sperm with structural abnormalities.***A*, photo of WT and *Stk33*^*-/KI*^ testes. *B*, average testis weight/body weight in WT and *Stk33*^*-/KI*^ mice. (N = 5). *C*, average number of pups per litter from WT and *Stk33*^*-/KI*^ mice. (N = 4). *D*, sperm count in single cauda epididymis from WT and *Stk33*^*-/KI*^ mice. (N = 4). *E*-*F*, average percentages of motile (*E*) and progressively motile (*F*) spermatozoa from WT and *Stk33*^*-/KI*^ mice. (N = 6). *G*, Western blot assay indicated that *Stk33*^*-/KI*^ testes expressed truncated STK33 protein. *H*, H&E staining of spermatozoa from WT and *Stk33*^*-/KI*^ mice. The scale bar represents 10 μm. *I*, the percentage of abnormal sperm heads and tails in WT and *Stk33*^*-/KI*^ mice. (N = 4). *J*, PAS staining of testis sections from adult WT and *Stk33*^*-/KI*^ mice. The scale bar represents 10 μm. (*Red box* in the *upper right* corner: magnification of abnormal spermatids; the *red dotted line* indicated decrease of spermatozooon flagella in stage VII-VIII seminiferous tubules of *Stk33*^*-/KI*^ mice). *K*, ultrastructure of cross sections of sperm head, middle piece, and principal piece from *Stk33*^*-/KI*^ mice. Abnormal heads and flagella are indicated by *arrowheads*. *Pink arrowheads* represent abnormal heads; *green arrowheads* represent abnormal mitochondria; *yellow arrowheads* represent abnormal outer dense fibers; *blue arrowheads* represent abnormal microtubules; and *orange arrowheads* represent abnormal fibrous sheaths (longitudinal columns and circumferential ribs). The scale bar represents 500 nm. All data are presented as mean ± SEM and analyzed by unpaired 2-tailed Student’s *t* test. NS, not significant; ∗*p*< 0.05, ∗∗∗*p*< 0.001. CR, circumferential ribs; D, diplotene; ES, elongated spermatids; FS, fibrous sheaths; L, leptotene; LC, longitudinal columns; N, nucleus; M, mitochondria; MT, microtubules; ODF, outer dense fibers; P, pachytene; PAS, periodic acid-Schiff; RS, round spermatids; Stk33, serine/threonine kinase 33; Z, Zygotene.

The frameshift mutation (*Stk33*^*−/−*^) and nonsense mutation (*Stk33*^*KI/KI*^) in mice both showed defects in male fertility but exhibited different levels of abnormalities in spermiogenesis and sperm morphologies. To further analyze the effects of compound frameshift and nonsense mutations in spermiogenesis and sperm structures, we generated *Stk33*^*-/KI*^ mice. *Stk33*^*-/KI*^ mice showed normal testicular appearance and size, and no change in the testis/body weight ratio compared to that of WT mice (Fig. 3, *A* and *B*). Intriguingly, *Stk33*^*-/KI*^ males were sterile when copulated with WT females (Fig. 3*C*). *Stk33*^*-/KI*^ males had reduced sperm count in cauda epididymis (Fig. 3*D*). The CASA assay indicated that *Stk33*^*-/KI*^ sperm were barely motile (supplemental Movie S2), with significantly decreased motility and progressive motility (Fig. 3, *E* and *F*). *Stk33*^*-/KI*^ mice testis also expressed truncated STK33 protein (Fig. 3*G*). Morphological analysis of *Stk33*^*-/KI*^ sperm showed malformation in 36.4% of heads and 59.2% of tails with short, curled, and missing flagella (Fig. 3, *H* and *I*). According to PAS staining, the head of *Stk33*^*-/KI*^-elongated spermatids appeared deformed during IX-X stages, and the flagella of *Stk33*^*-/KI*^ spermatids were shortened and scattered with the reduced amount at IV-VIII stages, which were similar to those of *Stk33*^*−/−*^-elongated spermatids (Fig. 3*J*). We performed an ultrastructural analysis of *Stk33*^*-/KI*^ sperm by transmission electron microscope. As a result, *Stk33*^*-/KI*^ sperm displayed nuclear malformation and structural abnormalities of flagella, including misarrangement of mitochondria and fibrous sheath, partial deletion of outer dense fiber, and deletion of microtubules in axoneme (Fig. 3*K*). Thus, *Stk33*^*-/KI*^ mice exhibited a sterile phenotype with reduced number and malformed structure of sperm.

### STK33 Kinase Regulates the Phosphoproteome Involved in Spermiogenesis

**Fig. 4:**
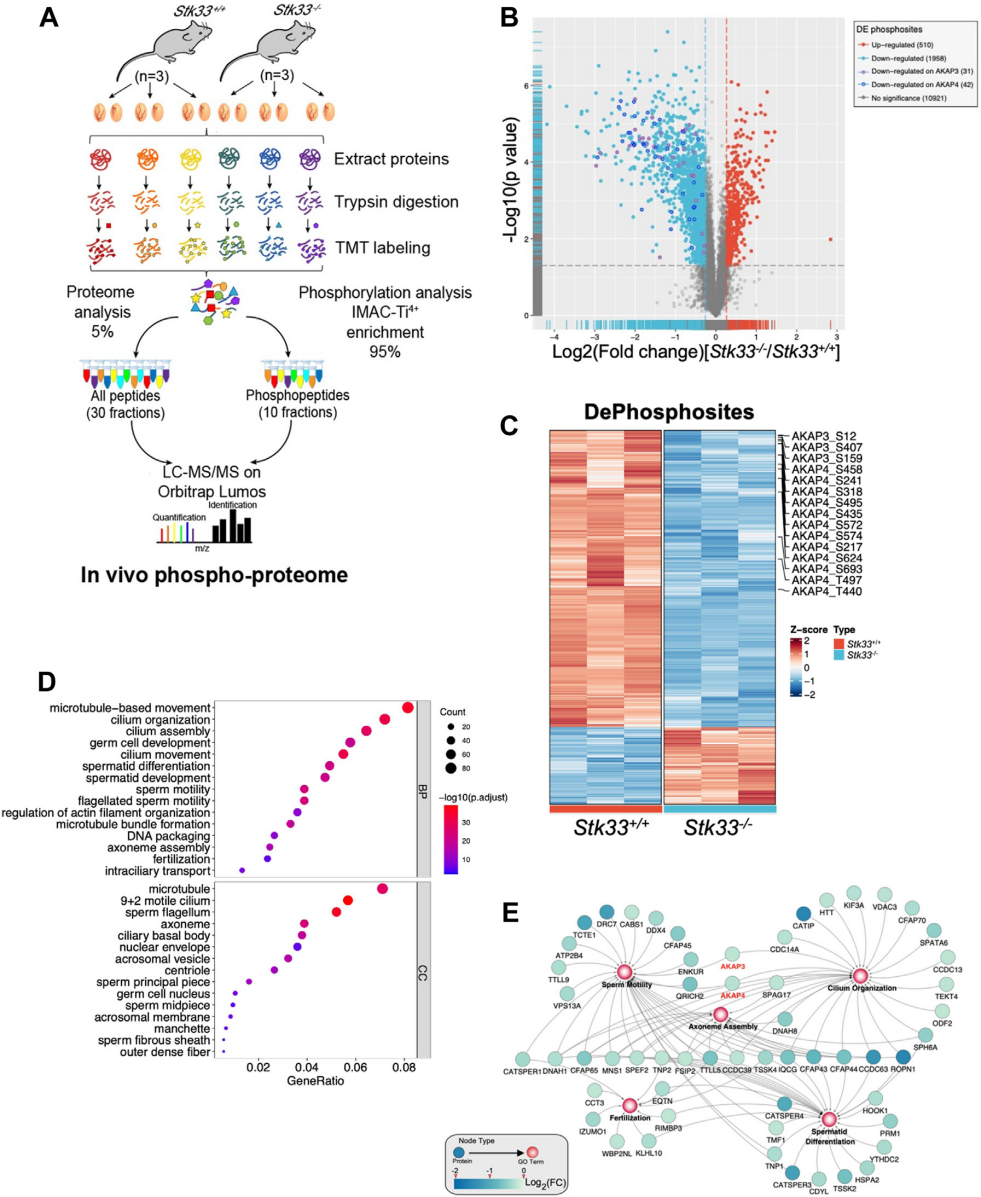
**Phosphoproteomic profiling of *Stk33***^***−/−***^**testicular proteins.***A*, schematic diagram illustrating differential phosphoproteomic workflow. *B*, volcano plot for the comparison of quantified protein phosphorylation sites between the WT and *Stk33*^*−/−*^ mice. The cutoff values (localization probability > 0.75, fold change > 1.2, and *p* < 0.05) were utilized to show phosphorylation sites with differential levels. Unchanged phosphorylation sites were shown in *gray*. The *blue and red dots* indicate downregulated and upregulated phosphosites, respectively. The *purple- and blue-circled dots* indicate downregulated phosphosites on AKAP3 and AKAP4, respectively. *C*, heat map plot of differentially regulated phosphorylation sites. *D*, gene ontology annotations of the proteins with downregulated phosphorylation levels in *Stk33*^*−/−*^ testes. Enriched terms of biological process and cellular component are shown as a bar graph. *E*, interaction network analysis of biological process terms constructed by Cytoscape. AKAP, A-kinase anchoring protein; Stk33, serine/threonine kinase 33.

As a kinase, STK33 was most likely to exert its functions *via* regulating protein phosphorylation. In order to characterize the regulatory roles of STK33 in spermiogenesis at the molecular level, we performed a phosphoproteomic analysis of *Stk33*^*−/−*^ testis after *Stk33* deletion (Fig. 4*A*). Among the identified 13,389 phosphosites in phosphoproteome, 2468 type I phosphosites (localization probability > 0.75, fold change > 1.2, and *p* < 0.05) were differentially regulated after normalization against protein expression levels, with 1958 (79.3%) phosphosites decreased and 510 (20.7%) phosphosites increased, corresponding to 1112 and 380 proteins, respectively (Fig. 4, *B* and *C*) (supplemental Table S1). The high percentage of downregulated phosphosites in *Stk33*^*−/−*^ testis indicated important roles in phosphorylation regulation by *Stk33* in testis. GO biological process analysis of proteins with downregulated phosphorylation levels showed enrichments in cilium organization (76 proteins), spermatid differentiation (52 proteins), sperm motility (41 proteins), axoneme assembly (26 proteins), and fertilization (25 proteins). Further annotation of GO cellular component terms displayed that the enriched terms related to “9 + 2” motile cilium (60 proteins), sperm flagellum (55 proteins), and axoneme (41 proteins) (Fig. 4, *D* and *E* and supplemental Table S2). The proteins with aberrantly downregulated phosphorylation are closely related to spermiogenesis to form sperm flagella structure, consistent with the deformities of sperm flagella after *Stk33* deletion.

### Mutations of *Stk33* Led to Defects of Its Substrate Fibrous Sheath Components AKAP3 and AKAP4

**Fig. 5:**
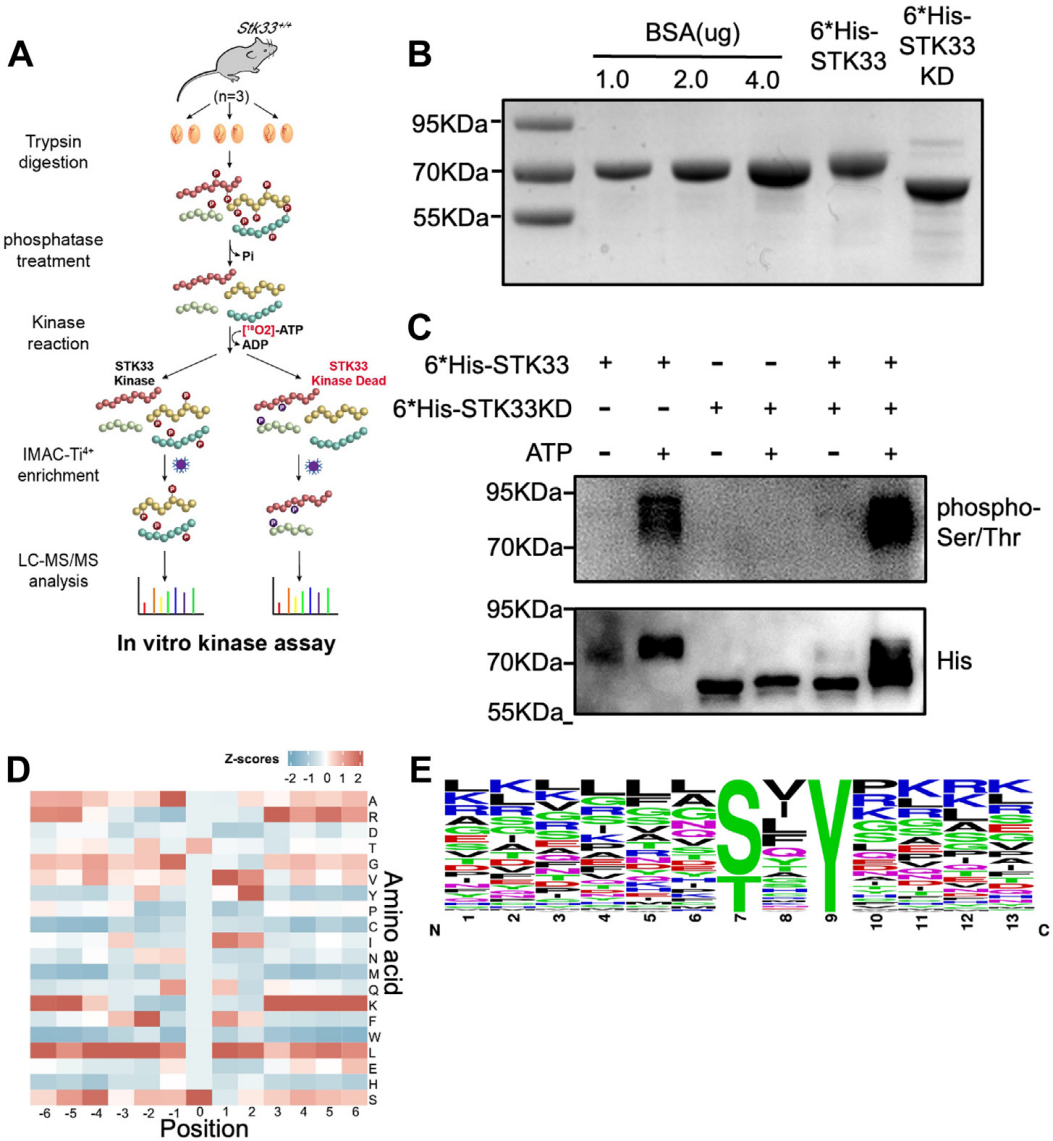
**Characterization of downstream substrates of STK33 kinase by *in vitro* kinase assay.***A*, schematic diagram of *in vitro* kinase assay. γ-[^18^O_4_] ATP and kinase-dead STK33 (K140M) (STK33 KD) were introduced to enhance sensitivity of reaction. *B*, prokaryotic expression of STK33 protein (6∗His-STK33) and STK33 KD protein (6∗His-STK33 KD). *C*, *in vitro* kinase assay using 6∗His-STK33 and 6∗His-STK33 KD proteins with or without ATP. STK33 kinase can phosphorylate itself, but STK33 KD failed to phosphorylate itself in the presence of ATP. *D* and *E*, motif analysis of sequences surrounding substrate phosphorylation sites of STK33 kinase by *in vitro* kinase assay; the distribution of amino acid residues is depicted by heat map (*D*) and by Motif-X (*E*). Stk33, serine/threonine kinase 33.

As various kinases act in the signaling cascade (21), the downregulated phosphorylation sites in *Stk33*^*−/−*^ testis might not all be direct substrates of STK33. To identify the direct phosphorylation substrates of STK33, we performed *in vitro* kinase assay of testicular peptides using purified STK33 protein. To avoid the interference of testis tissue–derived kinases, which may also have kinase activity in the reaction buffer, we chose to perform *in vitro* kinase assay at the peptide level instead of the protein level. Before *in vitro* kinase assay, the peptides from trypsin digestion of testicular proteins were treated by alkaline phosphatase to remove all phosphate groups to minimize the disturbance of residual peptidyl phosphates. To avoid the confounding effects of possible residual phosphopeptides due to incomplete dephosphorylation by alkaline phosphatase and increase the sensitivity of phosphorylation substrate identification, γ-[^18^O_4_] ATP was used to introduce ^18^O-labeled phosphate group in substrate phosphopeptides during *in vitro* kinase assay. The kinase-dead STK33 (K140M) with a mutation disrupting the functional kinase domain was used as a negative control (Fig. 5*A*). STK33 and STK33 (K140M) recombinant proteins were overexpressed in *E. coli* and purified by Ni-NTA column (Fig. 5*B*). We evaluated the kinase activities by *in vitro* kinase assay. Western blot analysis using an anti-phospho-serine/threonine antibody showed that STK33 could phosphorylate itself by self-phosphorylation, which could be stopped by the removal of ATP. As expected, there was no self-phosphorylation activity for kinase-dead STK33 (K140M) protein (Fig. 5*C*). Using the recombinant STK33 and STK33 (K140M) proteins, *in vitro* kinase assay followed by quantitative phosphoproteomics analysis identified a total of 7538 phosphosites, and 3748 type I phosphosites (localization probability > 0.75, fold change >10, and *p* < 0.05) were upregulated and were potential substrate phosphosites of STK33 kinase (supplemental Table S3). Analysis of the distribution of amino acids surrounding the phosphorylation sites showed that alanine was enriched at position −1 and valine and leucine were enriched at positions +1, respectively (Fig. 5*D*), and motif analysis using Motif-X revealed that (pS)xY (adjusted *p* = 1.6E-51) and (pT)xY (adjusted *p* = 5E-24) were the most enriched two motifs (Fig. 5*E*). Thus, STK33 is an active kinase with a wide spectrum of substrate phosphorylation sites.

**Fig. 6:**
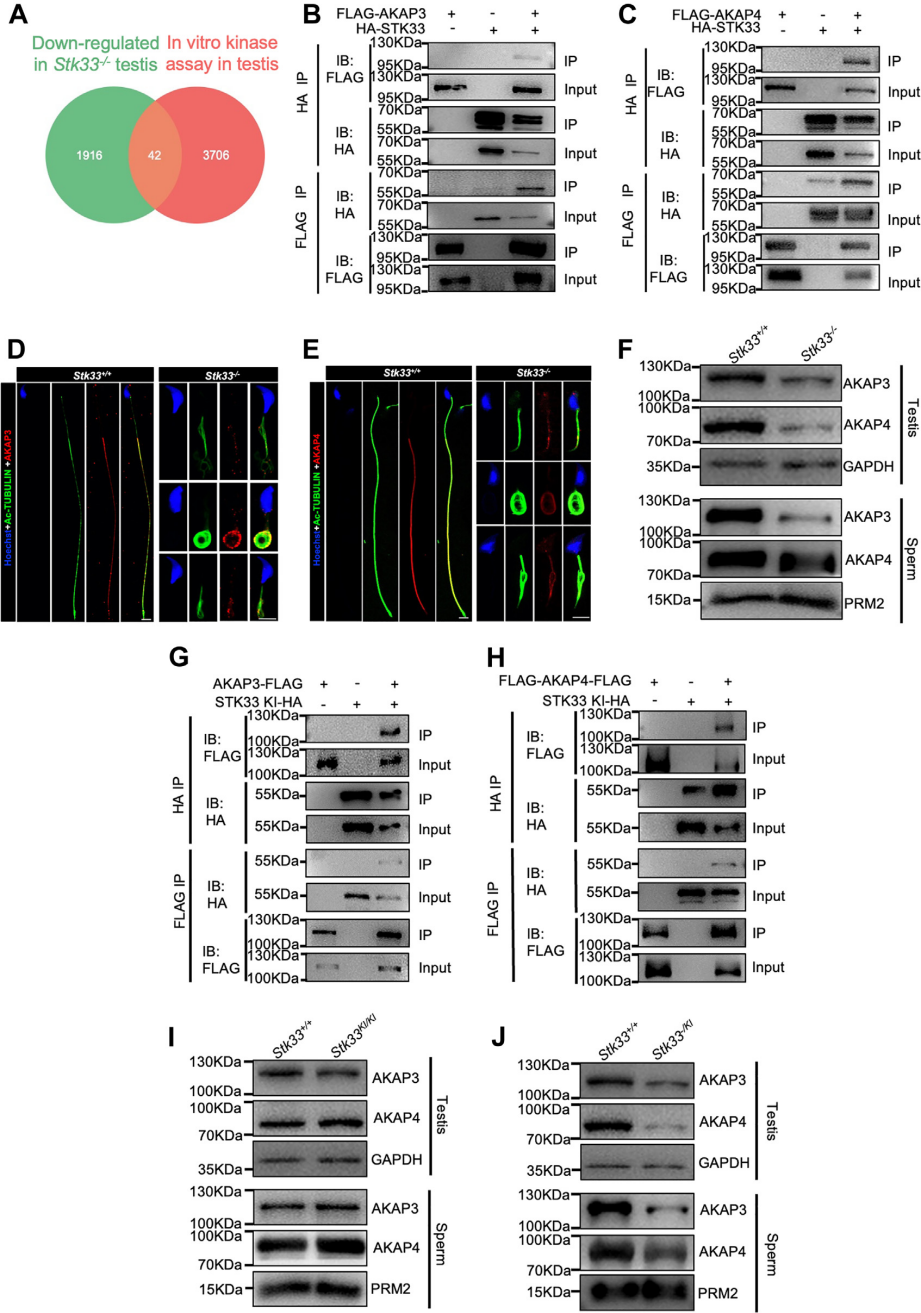
**Fibrous sheath proteins AKAP3 and AKAP4 are downstream substrates of STK33 kinase.***A*, Venn diagram illustrating the overlap of the downregulated phosphosites in *Stk33*^*−/−*^ testis and the substrate phosphosites by *in vitro* kinase assay at peptide level. The overlap represents STK33 substrates downregulated after deletion of Stk33. *B*, AKAP3-FLAG was co-expressed with STK33-HA in HEK293T cells, and interaction between STK33-HA and AKAP3-FLAG was examined by reciprocal co-immunoprecipitation. *C*, FLAG-AKAP4-FLAG was co-expressed with STK33-HA in HEK293T cells, and interaction between STK33-HA and FLAG-AKAP4-FLAG was examined by reciprocal co-immunoprecipitation. *D* and *E*, immunofluorescence staining of AKAP3/4 (*red*) and acetylated (Ac)-TUBULIN (*green*) in sperm from WT and *Stk33*^*−/−*^mice. The scale bar represents 5 μm. *F*, Western blot analysis of AKAP3 and AKAP4 proteins in *Stk33*^*−/−*^ testis and sperm; GAPDH and PRM2 served as loading controls. *G*, AKAP3-FLAG was co-expressed with STK33 KI-HA in HEK293T cells, and interaction between STK33 KI-HA and AKAP3-FLAG was examined by co-immunoprecipitation. *H*, FLAG-AKAP4-FLAG was co-expressed with STK33 KI-HA in HEK293T cells, and interaction between STK33 KI-HA and FLGA-AKAP4-FLAG was examined by co-immunoprecipitation. *I*, Western blot analysis of AKAP3 and AKAP4 proteins in *Stk33*^*KI/KI*^ testis and sperm; GAPDH and PRM2 served as loading controls. *J*, Western blot analysis of AKAP3 and AKAP4 proteins in *Stk33*^*-/KI*^ testis and sperm; GAPDH and PRM2 served as loading controls. AKAP, A-kinase anchoring protein; PRM2, Protamine 2; Stk33, serine/threonine kinase 33.

To identify the downregulated substrates of STK33 involved in spermiogenesis after *Stk33* deletion, we compared the substrate phosphorylation sites identified by *in vitro* kinase assay and the differential testicular phosphoproteome. We found that 42 phosphosites, corresponding to 29 phosphoproteins, were downregulated in *Stk33*^*−/−*^ testis and could be phosphorylated by recombinant STK33 *in vitro* (Fig. 6*A*). For fibrous sheath component AKAP3, 31 phosphorylation sites were downregulated in *Stk33*^*−/−*^ mouse testis, and three of these phosphosites were identified to be phosphorylated by STK33 in the *in vitro* kinase assay (supplemental Fig. S4*A*, supplemental Tables S1 and S3). Likewise, AKAP4 had 42 phosphorylation sites downregulated in *Stk33*^*−/−*^ mouse testis, and 12 of these phosphosites were identified to be phosphorylated by STK33 in the *in vitro* kinase assay (supplemental Fig. S4*B*, supplemental Tables S1 and S3). In addition, in the quantitative proteomic analysis of the testes from *Stk33*^*+/+*^ and *Stk33*^*−/−*^ mice, we identified a total of 10,266 proteins, 434 of which were differentially expressed proteins, including 263 upregulated proteins and 171 downregulated proteins in *Stk33*^*−/−*^ testis (fold change > 1.2 and *p* < 0.05) (supplemental Fig. S4, *C* and *D* and supplemental Table S4). Interestingly, the protein expression levels of AKAP3 and AKAP4, substrates of STK33, were significantly downregulated in *Stk33*^*−/−*^ testis. To analyze the interaction between STK33 and AKAP3/4, we performed a co-immunoprecipitation (Co-IP) assay by overexpressing AKAP3-FLAG/FLAG-AKAP4-FLAG and STK33-HA in HEK293T cells and confirmed the direct interaction between STK33 and AKAP3/4 (Fig. 6, *B* and *C*). Deletion of AKAP3 led to partially formed fibrous sheath with loss of circumferential ribs (22), while deletion of AKAP4 caused complete loss of fibrous sheath (23, 24), suggesting that the phosphorylation substrates AKAP3/4 of STK33 might mediate the defects in fibrous sheath formation after STK33 deletion.

Subsequently, to further evaluate the roles of AKAP3/4 after deletion of *Stk33*, we performed immunofluorescence and Western blot analysis of AKAP3/4 in *Stk33*^*−/−*^ mice. The immunofluorescence results showed that AKAP3 and AKAP4 were restricted in the principal piece of the WT sperm flagella, but both were lowly expressed in the flagellum of *Stk33*^*−/−*^ sperm (Fig. 6, *D* and *E*). The Western blot analyses showed that AKAP3 and AKAP4 were both downregulated in *Stk33*^*−/−*^ testis and sperm (Fig. 6*F*). Thus, STK33 deletion led to decreased expression of AKAP3/4, the major components of fibrous sheath.

In the *Stk33*^*KI/KI*^ mice, STK33 was expressed in a truncated form (STK33 KI). Thus, we further performed a Co-IP analysis of STK33 KI and AKAP3/4 and found that C-terminal truncation of STK33 in STK33 KI did not affect its interaction with AKAP3/4 (Fig. 6, *G* and *H*). However, unlike the *Stk33*^*−/−*^ mice, expressions of AKAP3/4 in *Stk33*^*KI/KI*^ mouse testes and sperm were almost the same as those in WT controls (Fig. 6*I*). As *Stk33*^*-/KI*^ mice were sterile with sperm flagella abnormalities, we further analyzed the expression of AKAP3/4 in *Stk33*^*-/KI*^ testis and sperm. The expression levels of STK33-/KI and AKAP3/4 in *Stk33*^*-/KI*^ mice showed a significant reduction compared to those of WT testes and sperm (Fig. 6*J*), which might be involved in the sperm flagella defects in *Stk33*^*-/KI*^ mice.

### Deletion of *Stk33* Led to Failure of Sperm to Induce Oocyte Activation

**Fig. 7:**
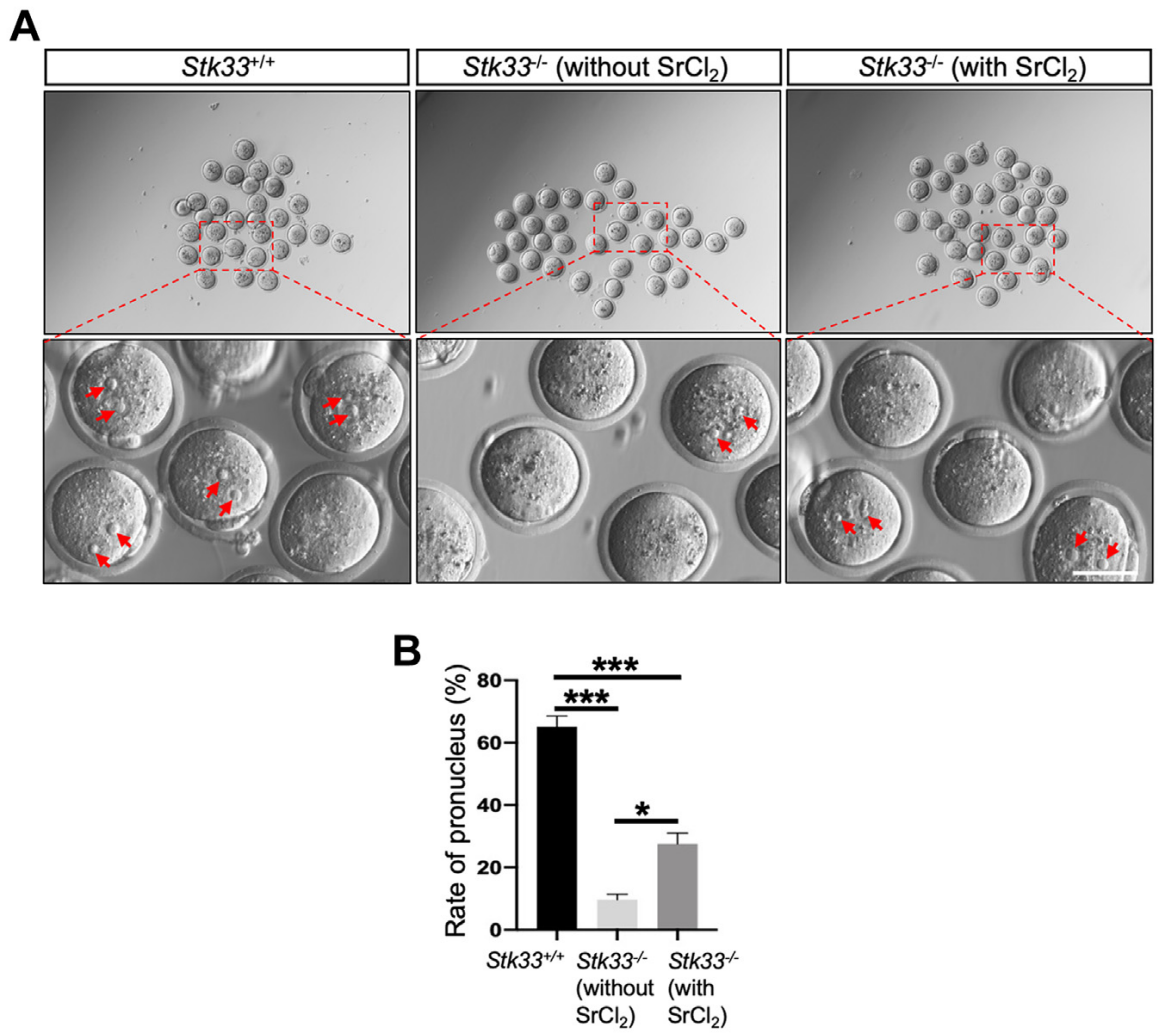
**Oocyte activation failure of sperm after *Stk33* deletion.***A*, the oocytes were *in vitro* fertilized by sperm from WT and *Stk33*^*−/−*^ mice with or without SrCl_2_ artificial activation. *Red arrowheads* indicate pronuclei of fertilized eggs. The scale bar represents 50 μm. *B*, statistics of pronucleus rate of WT and *Stk33*^*−/−*^ group with or without SrCl_2_ artificial activation. (N = 3). All data are presented as mean ± SEM and analyzed by one-way ANOVA. ∗*p* < 0.05, ∗∗∗*p* < 0.001. Stk33, serine/threonine kinase 33; SrCl2, strontium chloride.

GO biological process analysis of proteins with downregulated phosphorylation levels in *Stk33*^*−/−*^ testis showed enrichment proteins in fertilization, suggesting potential roles of STK33 in fertilization. To evaluate the function of *Stk33*^*−/−*^ sperm in fertilization, we performed an intracytoplasmic sperm injection (ICSI) assay (Fig. 7*A*). We found that the rate of pronucleus formation was significantly reduced from 65.1% in the WT sperm group to 9.7% in the *Stk33*^*−/−*^ sperm group 6 h after ICSI. Oocyte activation deficiency was known to be associated with ICSI failure (25), which could be improved by artificial oocyte activation (AOA). Hence, we checked whether the fertilization defect of *Stk33*^*−/−*^ sperm could be meliorated by AOA. After incubation with strontium chloride (SrCl_2_), a classical chemical agonist used to activate oocytes in ICSI, the pronucleus rates (27.6% *versus* 9.7%, *p* < 0.01) of the *Stk33*^*−/−*^ sperm group significantly increased compared to those without AOA treatment, indicating partial compensation of fertilization rate (Fig. 7*B*). STK33 is important for fertilization and oocyte activation induction.

## Discussion

Spermatogenesis abnormalities can lead to male infertility. It involves the regulation of testis-abundant genes, and the mechanisms are still not elucidated. Here, we identified nonsense (p.Glu446∗) and frameshift (p.Val170fs) mutations of *Stk33* in NOA. We generated *Stk33*^*KI/KI*^, *Stk33*^*−/−*^*,* and *Stk33*^*-/KI*^ mice to mimic the nonsense and frameshift mutations of *STK33* and found that they all had defects in male fertility in mice. Mutations of *Stk33* (*Stk33*^*-/KI*^) caused male sterility and abnormalities in spermiogenesis and sperm flagella formation. Quantitative phosphoproteomic analysis and *in vitro* kinase assay identified AKAP3 and AKAP4 as phosphorylation substrates of STK33, which are downregulated after STK33 deletion and are essential for fibrous sheath structure of sperm flagella.

We found that *STK33* is mutated in infertile patients with NOA, but *Stk33*^*-/KI*^ and *Stk33*^*−/−*^ mice were sterile with decreased number of sperm with oligoasthenoteratozoospermia, and *Stk33*^*KI/KI*^ mice were subfertile with decreased number of sperm and asthenozoospermia. *STK33* has been found to have mutations in patients with asthenozoospermia in Pakistan (26). The phenotype variations between mice and humans might be attributed to humans’ complex genetic differences and environmental factors (19, 20, 27, 28). Environmental factors might also interact with genetic mutations, leading to more severe human phenotypes (29). Discrepancies of phenotypes between mice and humans have been reported. For example, deletion of *SPINK2*, an azoospermia-associated gene, caused oligo-teratozoospermia in mice (30), and knockout of *Wdr63*, a gene mutated in both multiple morphological abnormalities of the sperm flagella and NOA patients, led to infertility with decreased sperm number and abnormal flagellar morphology in mice (31). Therefore, it is reasonable to observe mutations of *STK33* in NOA patients, and its mutations led to asthenozoospermia or oligoasthenoteratozoospermia in mice.

STK33 is a testis-abundant kinase. Its downstream regulation of phosphorylation is still not known in testis. Our quantitative phosphoproteomic analysis of *Stk33*^*−/−*^ mouse testis revealed that 79.3% of the 2468 phosphosites with differential levels after *Stk33* deletion were downregulated. It seems that phosphorylation is mainly affected by *Stk33* deletion. GO term analysis of proteins with phosphorylation sites downregulated in *Stk33*^*−/−*^ testis revealed significant enrichment in cilia organization, sperm motility, skeleton protein assembly (such as microtubule bundle formation and axoneme assembly), and fertilization. STK33 kinase plays important roles in the phosphorylation regulation of sperm flagella assembly.

Our *in vitro* kinase assay identified AKAP3/4 as the phosphorylation substrates of STK33. Till now, only a few substrates of STK33 have been identified. For instance, vimentin is a substrate of STK33 and participates in photoperiod regulation in the endocrine system; ERK2 is another substrate of STK33 and regulates tyrosinemia and tyrosinemia-associated neurological disorders (32, 33). However, there is little connection between these known substrates and spermiogenesis or sperm flagella formation. Our identification of two novel substrates of STK33 in testis provides us molecular mechanism of its regulation of fibrous sheath formation. Deletion of *Stk33* led to downregulation of AKAP3/4 at both phosphorylation and protein expression levels in mice. And deletion of AKAP3 and AKAP4 were reported to cause defects in fibrous sheath formation (22, 23, 24), and AKAP3/4 may play important roles in mediating the function of STK33 in fibrous sheath formation. However, how the phosphorylation regulates the expression or function of AKAP3/4 during fibrous sheath formation still needs further studies.

In short, we found that the mutations of *Stk33* could lead to defects in male fertility and spermiogenesis. Deletion of *Stk33* led to downregulation of protein phosphorylation involved in sperm flagella formation. STK33 regulated the phosphorylation of its substrates, AKAP3 and AKAP4, fibrous sheath components, and the formation of fibrous sheath. Our results shed new light on our understanding of phosphorylation regulation in spermatogenesis and male infertility.

## Data availability

The mass spectrometry proteomics data have been deposited to the ProteomeXchange Consortium *via* the PRIDE (34) partner repository (https://www.ebi.ac.uk/pride/archive) with the dataset identifier PXD036903 and could be accessed *via* a reviewer account (Username: reviewer_pxd036903@ebi.ac.uk, password: aoGamOIu).

## Consent to participate

Each patient completed a written informed consent before taking part in this research.

## Supplemental data

This article contains supplemental data.

## Conflict of interest

The authors declare no competing interests.
