# Supplementary material for: STK33 Phosphorylates Fibrous Sheath Protein AKAP3/4 to Regulate Sperm Flagella Assembly in Spermiogenesis
Source: Mol Cell Proteomics. 2023 May 3;22(6):100564. doi: 10.1016/j.mcpro.2023.100564 (PMC10245119; doi:10.1016/j.mcpro.2023.100564)
Supplement: Supplemental information [file mmc6.pdf]

Supplementary Information for

STK33 phosphorylates fibrous sheath protein AKAP3/4 to regulate sperm flagella assembly in spermiogenesis

Weiling Yu[1,#], Yang Li[1,2,#], Hong Chen[1,#], Yiqiang Cui[1,#], Chenghao Situ[1,#], Liping Yao[1], Xiangzheng Zhang[1], Shuai Lu[1,2], Li Liu[1], Laihua Li[1], Jie Ren[1], Yueshuai Guo[1], Zian Huo[1], Yu Chen[1], Haojie Li[1], Tao Jiang[1,2], Yayun Gu[1,2], Cheng Wang[1,2], Tianyu Zhu[1], Yan Li[1], Zhibin Hu[1,2,*], Xuejiang Guo[1,*]

*Corresponding author. Email: guo_xuejiang@njmu.edu.cn (XG);
zhibin_hu@njmu.edu.cn (ZH)

[1]State Key Laboratory of Reproductive Medicine, Nanjing Medical University, Nanjing, 211166, China.

[2]School of Public Health, Center for Global Health, Nanjing Medical University, Nanjing, Jiangsu, 211100, China.

This Supplementary Information include:

Figs. S1 to S4

Tables S1 to S5

Movies S1 to S2

Figure S1

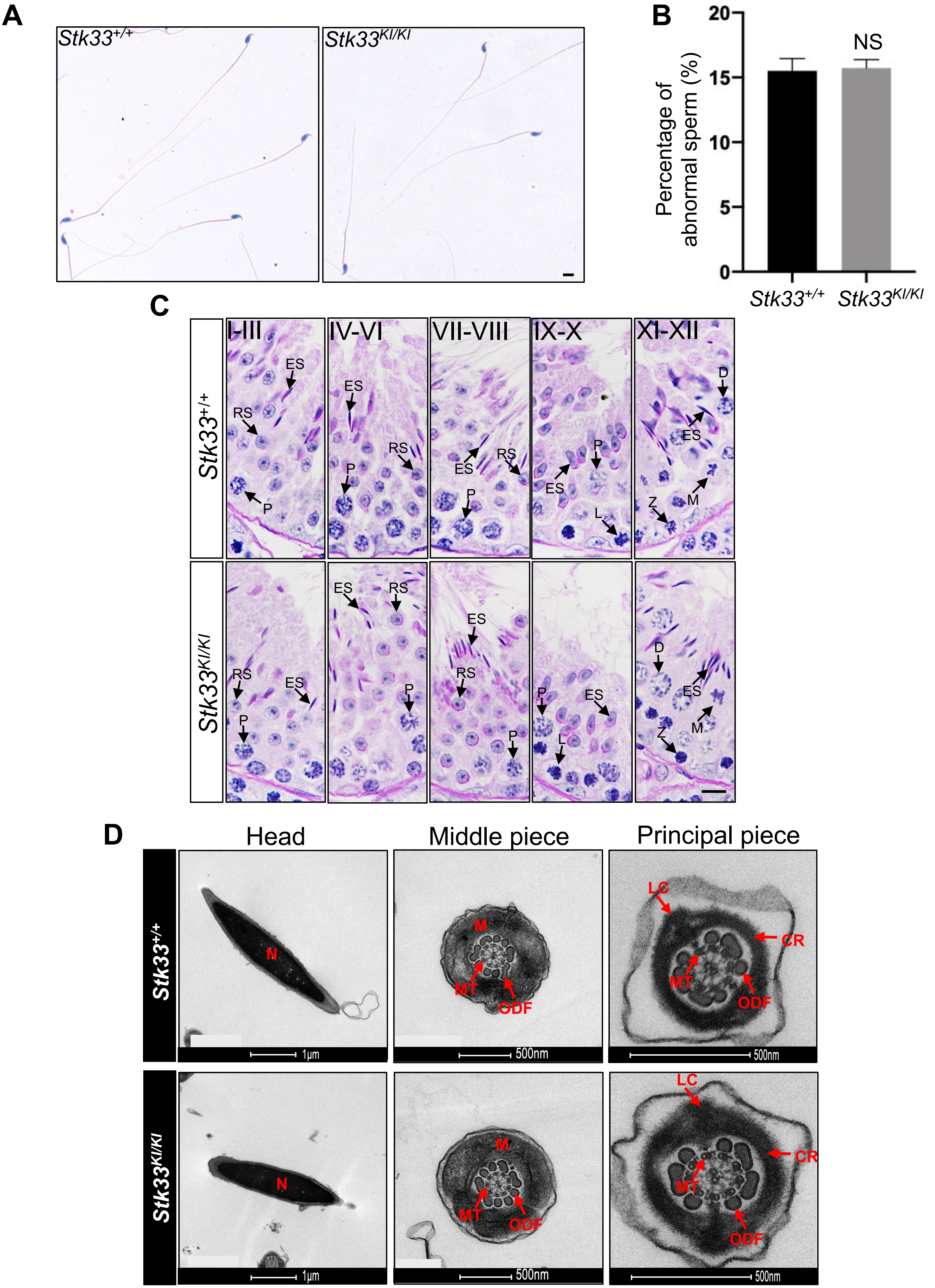
A
Stk33+/+
Stk33KI/KI
B
Percentage of abnormal sperm (%)
0
5
10
15
20
NS
Stk33+/+
Stk33KI/KI
C
I-III
IV-VI
VII-VIII
IX-X
XI-XII
Stk33+/+
Stk33KI/KI
ES
RS
P
L
D
Z
M
D
Head
Middle piece
Principal piece
N
M
MT
ODF
LC
CR
1μm
500nm

Figure S1. Phenotype analysis of wildtype and *Stk33*$^{KI/KI}$ mice.

(A) HE staining of spermatozoa from wildtype and *Stk33*$^{KI/KI}$ mice. Scale bar =10 μm. (B) Percentage of sperm abnormalities from wildtype and *Stk33*$^{KI/KI}$ mice. (N = 3). (C) PAS staining of testis sections from adult wildtype and *Stk33*$^{KI/KI}$ mice. Scale bar=10 μm. L, leptotene; Z, Zygotene; P, pachytene; D, diplotene; RS, round spermatids; ES, elongated spermatids; M, metaphase. (D) Ultrastructure of cross sections of sperm head, middle piece and principal piece of wildtype and *Stk33*$^{KI/KI}$ sperm. N, nucleus; M, mitochondria; MT, microtubules; ODF, outer dense fibers; LC, longitudinal columns; CR, circumferential ribs. Scare bar=500 nm/1 μm.

Figure S2

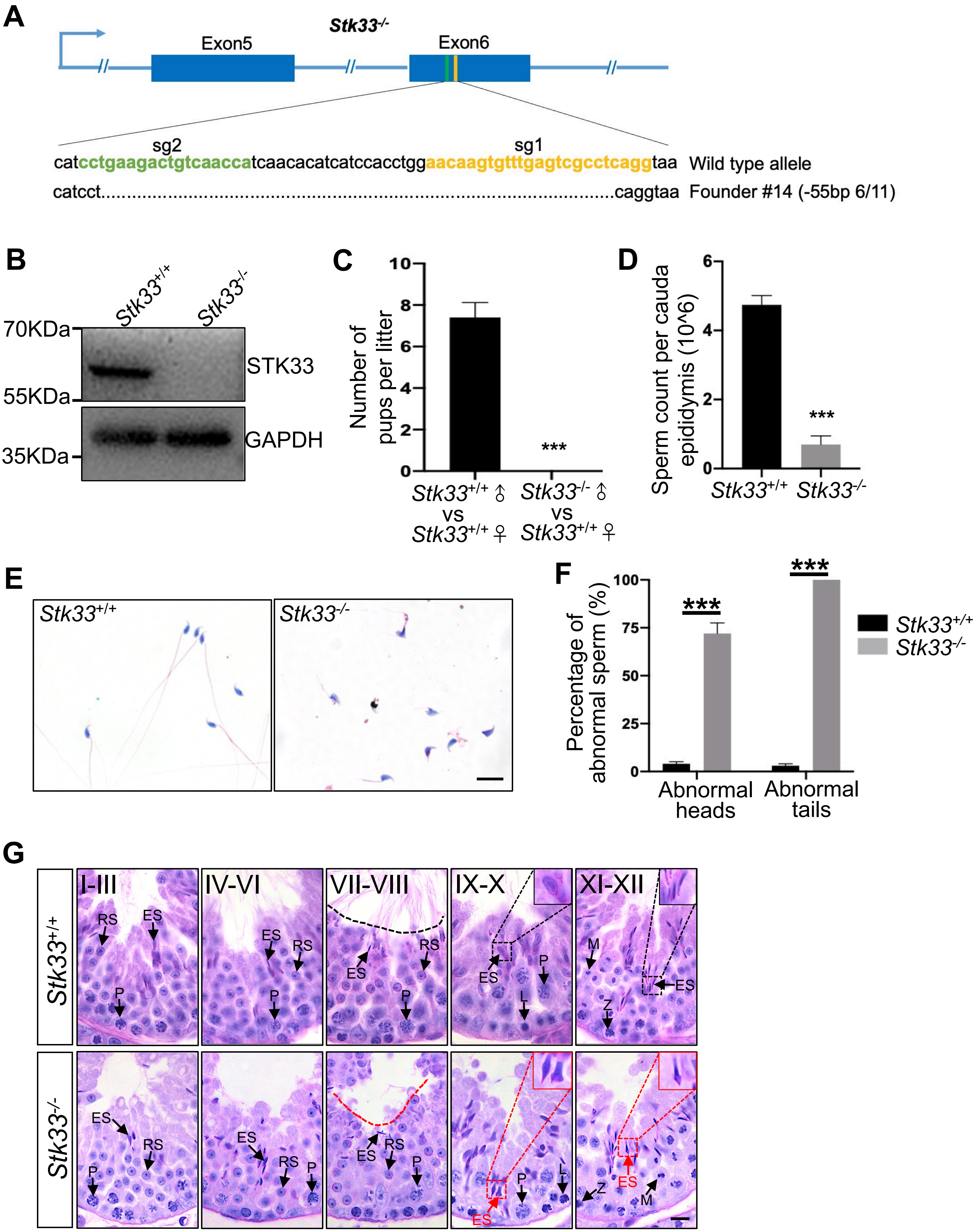
A
Stk33-/-
Exon5
Exon6
sg2
sg1
catcctgaagactgtcaaccatcaacacatcatccacctggaacaagtgtttgagtcgcctcaggtaa Wild type allele
catcct..........caggtaa Founder #14 (-55bp 6/11)
B
Stk33+/+
Stk33-/-
70KDa
55KDa
35KDa
STK33
GAPDH
C
Number of pups per litter
Stk33+/+ ♂ vs Stk33+/+ ♀
Stk33-/- ♂ vs Stk33+/+ ♀
***
D
Sperm count per cauda epididymis (10^6)
Stk33+/+
Stk33-/-
***
E
Stk33+/+
Stk33-/-
F
Percentage of abnormal sperm (%)
Abnormal heads
Abnormal tails
***
Stk33+/+
Stk33-/-
G
I-III
IV-VI
VII-VIII
IX-X
XI-XII
Stk33+/+
Stk33-/-
RS
ES
P
L
M
Z

Figure S2. Generation and phenotype analysis of male *Stk33*$^{-/-}$mice.

(A) Schematic diagram of the generation of *Stk33*$^{-/-}$ mice by Cas9 and sgRNAs. (B) Western blot analysis of STK33 in wildtype and *Stk33*$^{-/-}$testes with GAPDH as the loading control. (C) Average numbers of pups per litter from wildtype mice and *Stk33*$^{-/-}$male mice. (N = 5). (D) Sperm count in single cauda epididymis from wildtype mice and *Stk33*$^{-/-}$mice. (N = 3). (E) HE staining of spermatozoa from wildtype and *Stk33*$^{-/-}$ cauda epididymis. Scale bar=10 μm. (F) The percentage of abnormal sperm heads and tails in wildtype mice and *Stk33*$^{-/-}$mice. (N = 3). (G) PAS staining of testis sections from wildtype and *Stk33*$^{-/-}$mice. Scale bar=10 μm. (Red box in the upper right corner: magnification of abnormal spermatids; the red dotted line indicated decrease of spermatozoon flagella in stage VII-VIII seminiferous tubules of *Stk33*$^{-/-}$mice). L, leptotene; Z, Zygotene; P, pachytene; D, diplotene; RS, round spermatids; ES, elongated spermatids; M, metaphase.

Figure S3

A

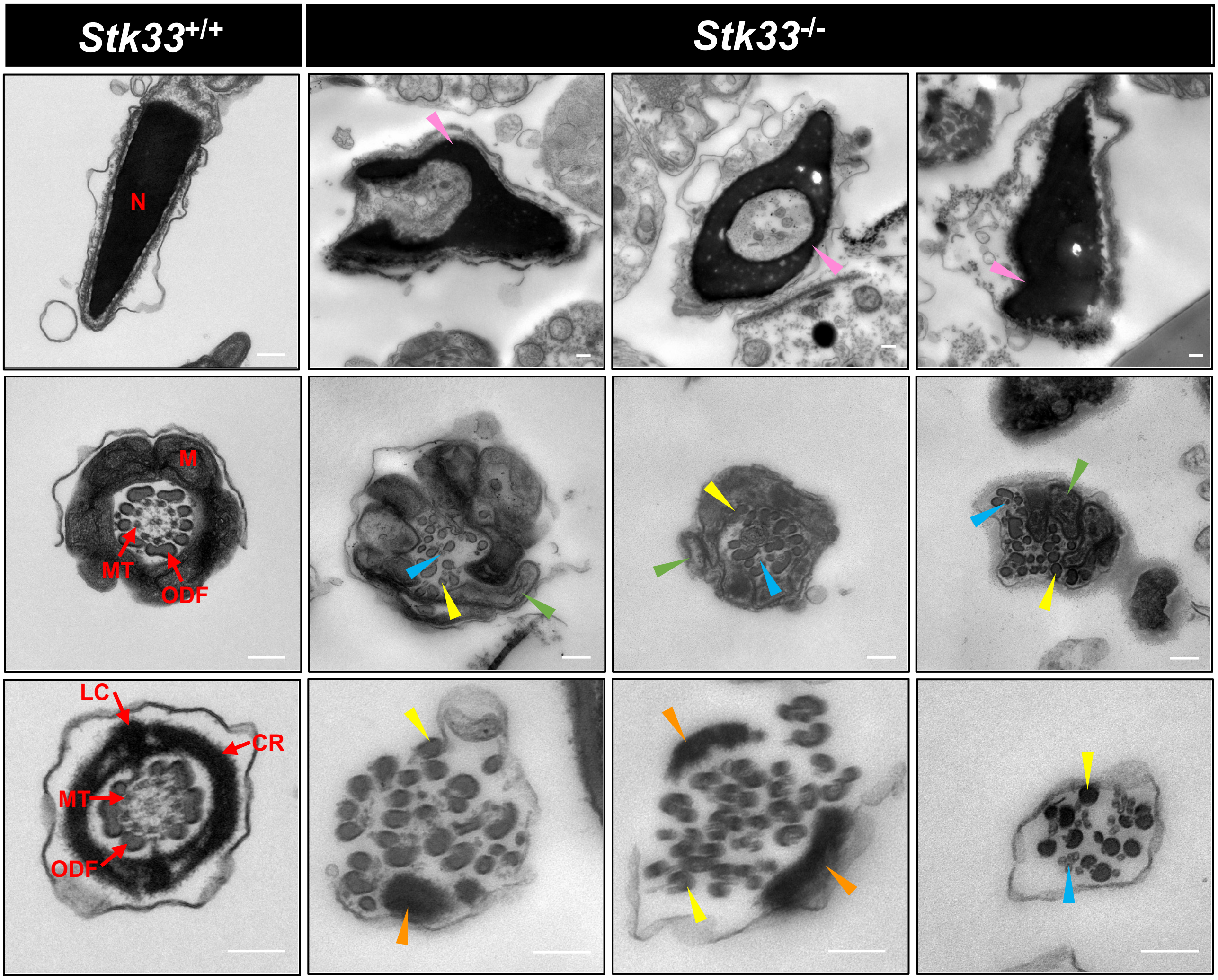

Stk33+/+
Stk33-/-
Head
Middle piece
Principal piece
N
M
MT
ODF
LC
CR
MT
ODF

Figure S3. Ultrastructure analysis of male *Stk33*$^{-/-}$mice.

(A) Ultrastructure of cross sections of sperm head, middle piece and principal piece from *Stk33*$^{-/-}$ mice showed abnormal head, disorganized arrangement of mitochondria and ODF, disrupted "9+2" microtubule, and incomplete fibrous sheath in the principal piece. Abnormal heads and flagella indicated by arrowheads. Pink arrowheads: abnormal heads; green arrowheads: abnormal mitochondria; yellow arrowheads: abnormal outer dense fibers; blue arrowheads: abnormal microtubules; orange arrowheads: abnormal fibrous sheaths (longitudinal columns and circumferential ribs). N, nucleus; M, mitochondria; MT, microtubules; ODF, outer dense fibers; LC, longitudinal columns; CR, circumferential ribs; FS, fibrous sheaths. Scare bar =200 nm.

Figure S4

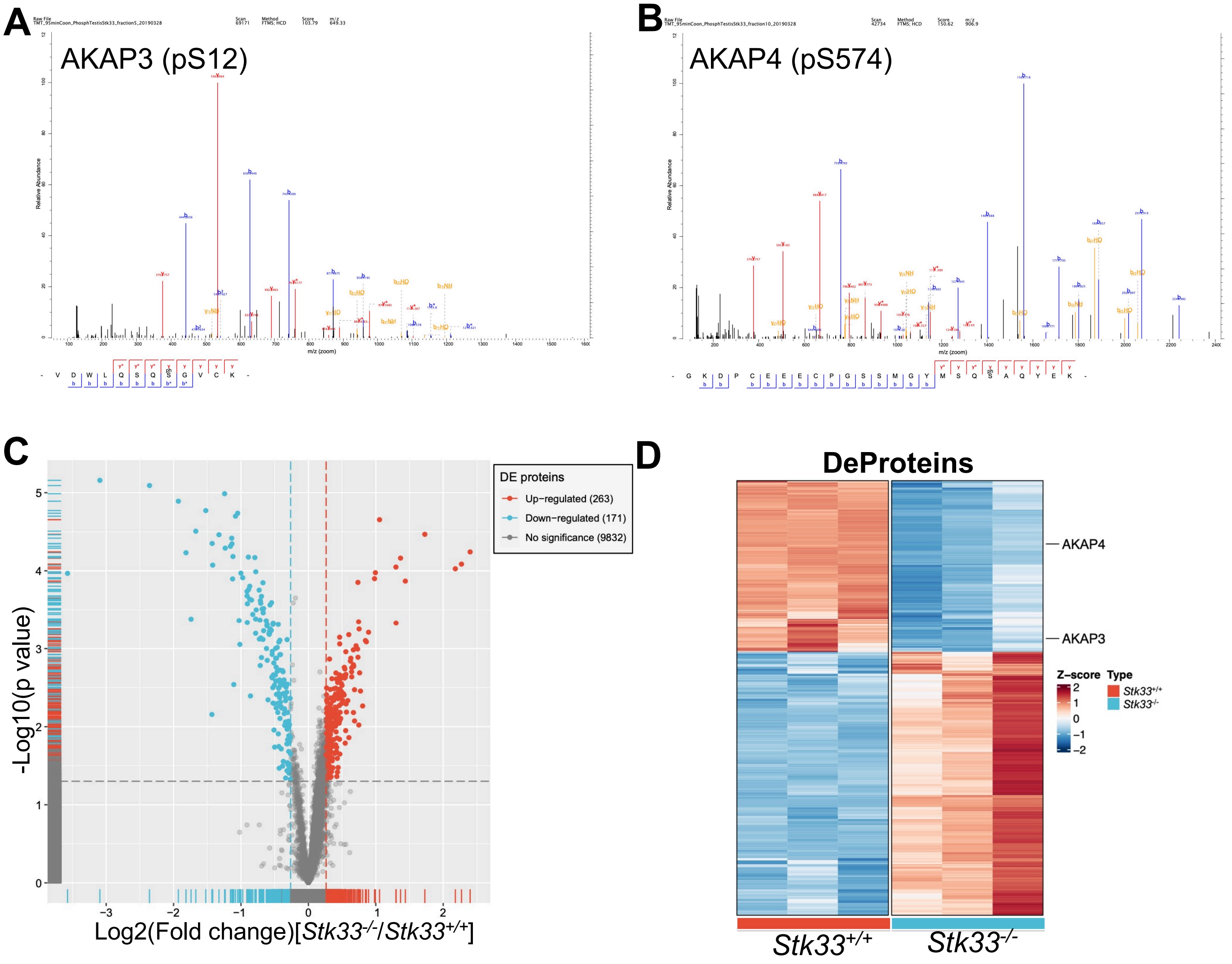
A
AKAP3 (pS12)
Relative Abundance
m/z (zoom)
B
AKAP4 (pS574)
C
DE proteins
Up−regulated (263)
Down−regulated (171)
No significance (9832)
-Log10(p value)
Log2(Fold change)[Stk33-/-/Stk33+/+]
D
DeProteins
AKAP4
AKAP3
Z−score
Type
Stk33+/+
Stk33-/-

Figure S4. Proteomic and phosphoproteomic analysis of *Stk33*$^{+/+}$ and *Stk33*$^{-/-}$ testes

(A) Volcano plot of quantified proteins between the wildtype and *Stk33*$^{-/-}$testes. The cutoff values (fold change > 1.2 and p< 0.05) were utilized to show differentially expressed proteins. Unchanged proteins were shown in grey. The blue and red dots indicate of down-regulated and up-regulated proteins, respectively. (B) Heatmap plot of differentially expressed proteins between the wildtype and *Stk33*$^{-/-}$testes. (C) AKAP3 (pS12) phosphopeptide sequence identified by MS/MS. m/z, Mass-to-charge. (D) AKAP4 (pS574) phosphopeptide sequence identified by MS/MS. m/z, Mass-to-charge.

Table S1. Differential phosphosites between *Stk33*$^{+/+}$ and *Stk33*$^{-/-}$ testes.

Table S2. Gene ontology enrichment analysis of proteins with down-regulated levels of phosphorylation in *Stk33*$^{-/-}$ testes.

Table S3. Up-regulated phosphosites identified by LC-MS/MS using Stk33 kinase by in vitro kinase assay.

Table S4. Differential proteins identified by LC-MS/MS between *Stk33*$^{+/+}$ and *Stk33*$^{-/-}$ testes.

Table S5. The primers used in this study.

Movie S1. Sperm from *Stk33*$^{+/+}$and *Stk33*$^{KI/KI}$ caudal epididymis. Video recording of sperm from *Stk33*$^{+/+}$and *Stk33*$^{KI/KI}$ caudal epididymis by the Computer Assisted Sperm Analyzer.

Movie S2. Sperm from *Stk33*$^{+/+}$and *Stk33*$^{-/KI}$ caudal epididymis. Video recording of sperm from *Stk33*$^{+/+}$and *Stk33*$^{-/KI}$ caudal epididymis by the Computer Assisted Sperm Analyzer.
